# Adhesive and Self-Healing Polyurethanes with Tunable Multifunctionality

**DOI:** 10.34133/2022/9795682

**Published:** 2022-10-26

**Authors:** Lei Zhou, Lu Zhang, Peichuang Li, Manfred F. Maitz, Kebing Wang, Tengda Shang, Sheng Dai, Yudie Fu, Yuancong Zhao, Zhilu Yang, Jin Wang, Xin Li

**Affiliations:** ^1^School of Materials Science and Engineering, Southwest Jiaotong University, Department of Cardiology, Third People's Hospital of Chengdu Affiliated to Southwest Jiaotong University, Chengdu, 610031 Sichuan, China; ^2^Heze Branch, Qilu University of Technology (Shandong Academy of Sciences), Biological Engineering Technology Innovation Center of Shandong Province, Heze 274000, China; ^3^Max Bergmann Center of Biomaterials Dresden, Leibniz Institute of Polymer Research Dresden, Hohe Strasse 6, 01069 Dresden, Germany; ^4^Affiliated Dongguan Hospital, Southern Medical University, Dongguan, Guangdong 523059, China; ^5^Guangdong Provincial Key Laboratory of Cardiac Function and Microcirculation, Guangzhou, Guangdong 510080, China

## Abstract

Many polyurethanes (PUs) are blood-contacting materials due to their good mechanical properties, fatigue resistance, cytocompatibility, biosafety, and relatively good hemocompatibility. Further functionalization of the PUs using chemical synthetic methods is especially attractive for expanding their applications. Herein, a series of catechol functionalized PU (C-PU-PTMEG) elastomers containing variable molecular weight of polytetramethylene ether glycol (PTMEG) soft segment are reported by stepwise polymerization and further introduction of catechol. Tailoring the molecular weight of PTMEG fragment enables a regulable catechol content, mobility of the chain segment, hydrogen bond and microphase separation of the C-PU-PTMEG elastomers, thus offering tunability of mechanical strength (such as breaking strength from 1.3 MPa to 5.7 MPa), adhesion, self-healing efficiency (from 14.9% to 96.7% within 2 hours), anticoagulant, antioxidation, anti-inflammatory properties and cellular growth behavior. As cardiovascular stent coatings, the C-PU-PTMEGs demonstrate enough flexibility to withstand deformation during the balloon dilation procedure. Of special importance is that the C-PU-PTMEG-coated surfaces show the ability to rapidly scavenge free radicals to maintain normal growth of endothelial cells, inhibit smooth muscle cell proliferation, mediate inflammatory response, and reduce thrombus formation. With the universality of surface adhesion and tunable multifunctionality, these novel C-PU-PTMEG elastomers should find potential usage in artificial heart valves and surface engineering of stents.

## 1. Introduction

For decades, many polyurethanes (PUs) have been extensively applied in the blood-contacting field because they can be tailored for excellent mechanical compliance, easy molding, and relatively good hemocompatibility [1–3], such as artificial heart valves [4], blood catheters [5], stent coatings [6], and artificial blood vessels [7]. However, with extended service time and broadened applications, the conventional PUs have suffered from some serious problems, such as degradation and loss of mechanical properties [2], adhesion of inflammatory cells [8], sedimentary protein [9], surface-induced thrombus formation, and inadequate endothelialization [10]. Although encouraging progress has been achieved in functionalized PUs prepared by various strategies to address these problems [2, 11], the lack of systemic tunability limits their wider application for blood-contacting devices. As developing new blood-contacting polymers for different applications is usually challenging and time-consuming [12], implementing PUs with tunable properties is of high practical and economic value.

Considering requirements for blood-contacting materials, low coagulation will be given the first priority, which is the cornerstone of normal operation for implants. Secondly, antioxidant and anti-inflammatory properties appear critical, which can stabilize the normal physiological microenvironment and facilitating tissue recovery at the implantation site [12, 13]. Since implants are easily damaged by mechanical and chemical external stresses to result in reduced service life and loss of function during their lifetime [14, 15], self-repairing properties can allow for functional recovery and prolong the implant life [16, 17]. In addition, extensive and adequate adhesion to the substrate, especially in wet media, is important to ensure the stable performance of implants [18], when PUs are used as a coating [19, 20]. Finally, in order to broaden the application field, flexible tunability of functions and properties (e.g. mechanical properties, cellular, and blood behavior) can help to adapt better to the requirements of the actual environment.

Various kinds of manners to functionalize the PU for hemocompatible applications have been suggested [21, 22], such as nitric oxide (NO) producing PUs [23], heparin-based PUs [3], phosphorylcholine, and/or fluorine-based PUs [24–26]. However, these materials are optimized for a single function with scarce tunability. Therefore, they are not widely applied in blood-contacting areas. In addition, developing various materials for different applications is highly time- and R&D cost-consuming.

Most PUs are block polymers composed of alternating hard and soft segments, synthesized by the reaction between diisocyanates, polyols, and chain extenders [27]. More than 500 commercially available monomers are chosen from [28], but it is extremely difficult to achieve multifunctional PUs using these monomers. Although multifunctionality may be achieved by appropriately functionalized monomers, their design and synthesis turned out to be a laborious and complicated process [29–31]. Hence, developing tunable multifunctional PU is a challenging goal for modifying various blood-contacting devices.

Materials with various properties such as adhesion and antioxidation have been developed using catechol and their derivatives [32–34] because of its multiple chemical properties. In this work, we proposed a simple strategy to obtain catechol functionalized PU (C-PU-PTMEG) elastomers with tunable multifunctionality to adapt to various blood applications. Through amide reaction, dopamine (DA) was introduced into carboxylated PUs (PU-PTMEGs), which contain controllable polytetramethylene ether glycol (PTMEG) fragment as soft segment, to acquire C-PU-PTMEGs. The key aspect of our PU elastomers design was to use the multiple properties of the catechol and its dynamic exchangeable hydrogen bonding with the chain segments of PU, which gave the PU multifunctionality (such as adhesion, self-healing ability, anti-coagulation, antioxidation, and anti-inflammation). More remarkably, these multifunctionalities and the properties of the PU (such as mechanical properties or cytocompatibility) could be controlled through variation of the PTMEG fragments. For example, the C-PU-PTMEGs could implement a high achievable adhesion of 2.7 MPa and complete repair within 2 hours by tuning the soft segment. Due to the tunable multifunctionality, we believe that these novel multifunctional C-PU-PTMEG elastomers with controlled mechanical properties, adhesion, self-healing, antioxidation, anti-inflammation, anticoagulation, and cellular behavior can be appropriate for a wide range of blood-contacting applications, such as artificial heart valves and surface engineering of stents.

## 2. Results and Discussion

### 2.1. Composition and Characterization of C-PU-PTMEGs

**Figure 1:**
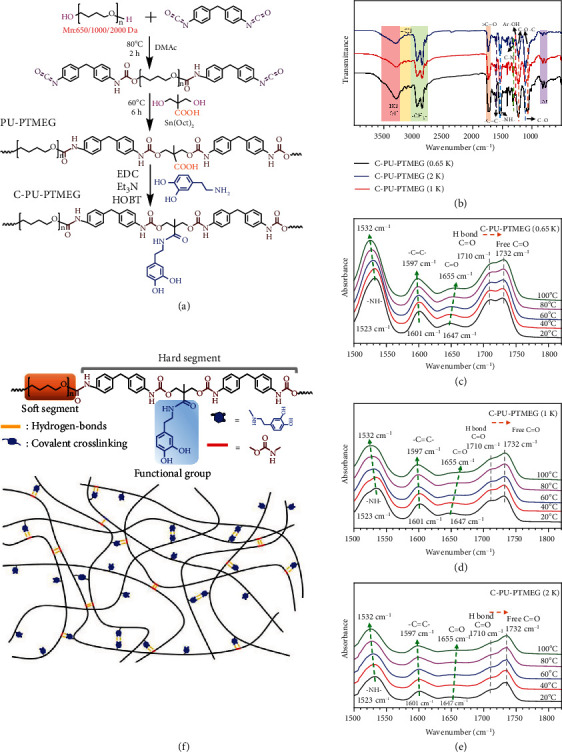
Chemical structure and FT-IR spectra of C-PU-PTMEGs. (a) Synthesis schematic and structures of PU-PTMEGs and C-PU-PTMEGs. (b) FT-IR spectra of C-PU-PTMEGs. Variable-temperature FI-TR spectra of (c) C-PU-PTMEG (0.65 K), (d) C-PU-PTMEG (1 K), and (e) C-PU-PTMEG (2 K) with an increasing temperature in the range of 1500 to 1850 cm^−1^. (f) Molecular structure of C-PU-PTMEGs and a schematic description of hydrogen bond moiety.

For adaptation to various blood-contacting applications, adjustable multifunctionality of the material is desired. Here, we implemented a simple strategy for the preparation of multifunctional PUs (synthesis route and monomer input as shown in Figure 1(a) and Table S1). Firstly, PU-PTMEGs (Figure S1A, Figure S2, and Table S2) were successfully obtained by prepolymerization and chain expansion through the reaction of PTMEG with diphenyl-methane-diisocyanate (MDI) and 2,2-Bis(hydroxymethyl) propionic acid (DMPA). Subsequently, the C-PU-PTMEGs were acquired by introducing the catechol into the structure of the PU-PTMEGs through amide reaction.

Fourier transform infrared (FT-IR) and ^1^H nuclear magnetic resonance (NMR) spectroscopy were used to verify the synthesis of the elastomers. As shown in Figure 1(b), the FT-IR spectrum displayed the diagnostic peaks of C-PU-PTMEGs, including the methylene, benzene ring of MDI and catechol, ether, carbamate, and phenolic hydroxyl groups. The absence of the -NCO peak (2240-2280 cm^−1^) indicated a complete polymerization reaction. The ^1^H NMR spectra of all inactive hydrogens were consistent with their theoretical chemical structures (Figure S1B). Further analysis of the FT-IR in 1680-1770 cm^−1^ region (Figure S3 and Table S4) and ^1^H NMR spectrum in the range of 6.2-7.5 ppm (Figure S1C) allowed to determine the degree of phase separation (DPS), catechol grafting rates, and contents, as shown in Table S3 and found that the DPS and catechol contents decreased with increasing molecular weight of the PTMEG from 0.65 to 2 kDa fragment (C-PU-PTMEG (0.65 K, 1 K, and 2 K)). Moreover, the catechol structure also increased the DPS of the PUs.

Gel permeation chromatography (GPC) was used to confirm the molecular weights of the synthesized elastomers, and all of them had molecular weights above 20 kDa (Table S2 and Table S3). In order to investigate the hydrogen bond formation in C-PU-PTMEGs, variable temperature FT-IR was performed. As shown in Figures 1(c)–1(e) and Figure S4, there were abundant hydrogen bonds, including bidentate hydrogen bonds (carbamate to carbamate, carbamate to phenolic hydroxyl, and phenolic hydroxyl to phenolic hydroxyl) and monodontic hydrogen bonds (carbamate to ether, phenolic hydroxyl to ether, and amine to phenyl [35, 36]). These results indicated that C-PU-PTMEGs were successfully synthesized and possessed an abundant variety of dynamic hydrogen bonding forms. The catechol content and microstructure of the PUs can be altered by modulating the PTMEG fragment.

**Figure 2:**
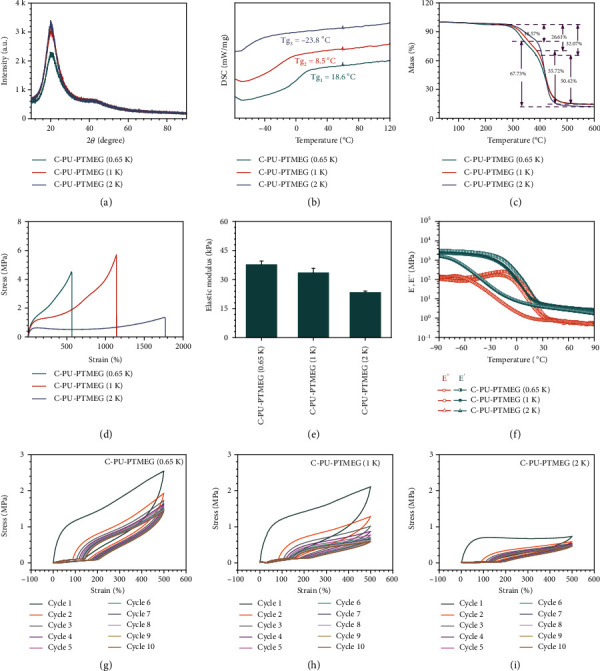
Characterization of C-PU-PTMEG polymers with different molecular weights of the PTMEG soft segment (0.65, 1, and 2 kDa). (a) XRD spectra. (b) DSC spectra in the range of -76 to 120°C. (c) TG within the scope of 25 to 600°C at a rate of 10°C min^−1^. (d) Tensile test and (e) its elastic modulus. (f) Temperature sweep of storage modulus and loss modulus. The successive loading-unloading cycles of (g) C-PU-PTMEG (0.65 K), (h) C-PU-PTMEG (1 K), and (i) C-PU-PTMEG (2 K) for 10 times at a tensile rate of 100 mm min^−1^.

Considering the regularity of the soft segment structure of the C-PU-PTMEGs, we presumed the existence of a crystalline structure, which may affect the mobility of the segment. X-ray diffraction (XRD) results were showed in Figure 2(a), indicating that the soft segments of all C-PU-PTMEGs had a semicrystalline structure. The intensity of the peaks reflected the degree of crystallinity and chain segment alignment [37]. Therefore, the increasing molecular weight of PTMEG segments favored the crystallization. Moreover, the rising hydrogen bonds and the cross-linking due to the catechol groups could reduce the crystallization of PU (Figure S5A) because of blocked chains.

The glass transition temperatures (Tg) of the C-PU-PTMEGs were dependent on the molecular weight of the PTMEG segment. Differential scanning calorimetry (DSC) results showed a decreasing Tg from 18.6 to -23.8°C (Figure 2(b)), revealing that C-PU-PTMEGs were in rubbery state when they served in the blood environment. Thermogravimetric (TG) curves (Figure 2(c)) revealed that the initial decomposition temperature of the C-PU-PTMEGs was about 274°C, related to the thermal decomposition of the hard segment, and the subsequent heating to near 388°C, where the soft segment started to decompose [38]. Thus, the C-PU-PTMEGs exhibited a good thermostability over a wide temperature range.

Subsequently, the mechanical properties of the C-PU-PTMEGs for blood-contacting materials were investigated. In the tensile tests, increasing the molecular weight of the PTMEG segment improved the tensile properties (Figure 2(d)) and reduced the elastic modulus of C-PU-PTMEGs (Figure 2(e)). Notably, it was found that the PTMEG segment with a molecular weight of 1 kDa had excellent comprehensive tensile properties, which could be stretched to 1138.7% with a fracture strength of 5.7 MPa. Figure 2(f) shows that the storage modulus (E′) of C-PU-PTMEGs was higher than the loss modulus (E^″^) during the whole tested process, indicating that C-PU-PTMEGs exhibited solid-like properties throughout the tested temperature range. The E′ of C-PU-PTMEGs decreased with increasing molecular weight of the PTMEG segment, revealing a reduced stiffness, which was consistent with the results of tensile tests. In addition, the decrease in E′ and E^″^ associated with the relaxation with the soft linking segment was significant, and three strong relaxation peaks were also present in the loss factors (tan*δ*), which were -28.4, 7.2, and 16.5°C, respectively (Figure S5B). The loss factor could be related to the Tg of the materials, similar to the DSC results, indicating sufficient chain mobility to reconstitute new hydrogen bonds at room temperature and *in vivo* environment.

Finally, the energy dissipation due to hydrogen bond formation was also evaluated by applying 10 cycles of 500% strain to the C-PU-PTMEGs. As shown in Figures 2(g)–2(i), the hysteresis occurred in each cycle and the C-PU-PTMEGs energy loss and elastic modulus decreased gradually during the successive loading-unloading cycles (Figure S6A and B), indicating the dissipation of hydrogen bond dissociation during the process of stretching. The lower molecular weight of the PTMEG fragment owned higher energy loss (Figure S6A) and elastic modulus of the C-PU-PTMEGs in every cycle point (Figure S6B). It was assumed that the PTMEG fragments with lower molecular weight had more hydrogen bonds due to the higher catechol content, increasing the physical cross-linkage of C-PU-PTMEGs, which required more hydrogen bonds to be broken when stretching the sample under the same conditions. At the same time, the increased physical cross-linkage improved the strength of the material. In general, the crystallinity, Tg, and mechanical properties of these thermostable C-PU-PTMEGs demonstrated a regular variation with a raising molecular weight of PTMEG segments.

### 2.2. Adhesion Properties

**Figure 3:**
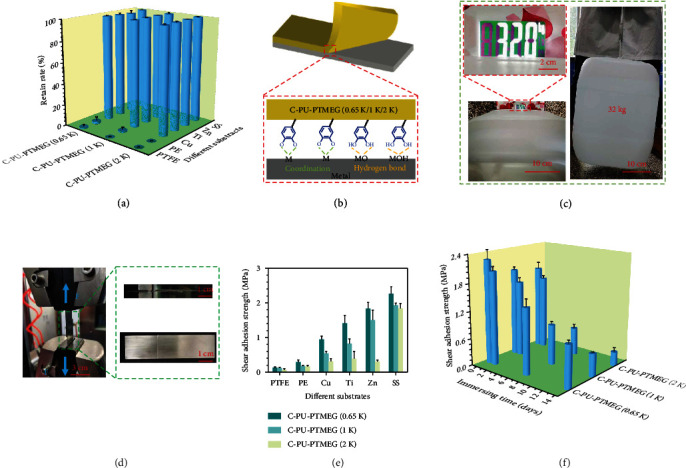
Adhesion tests. (a) The degree of retention of C-PU-PTMEGs with different molecular weight of the PTMEG component (0.65, 1, and 2 kDa) on the various material surfaces after scribing by special exact cutter and further adhering with 3 M tape for 5 min. (b) Schematic diagram of the adhesion mechanism of C-PU-PTMEGs on the surface of metallic materials. (c) Joint bonding of two pieces of 316L SS with a C-PU-PTMEG (1 K) film (40 *μ*m thick) for one day withstand a weight of 32 kg. (d) Photograph of the joints prepared with C-PU-PTMEGs and different materials being stretched by a universal stretching machine at a speed of 1 mm/min. (e) Shear adhesion strength of the three C-PU-PTMEGs on various surfaces in dry environment. (f) Shear adhesion strength of C-PU-PTMEGs in a wet environment by bonding of two pieces of 316L SS.

Excellent adhesion, especially in wet environments, is essential for a coating material of blood-contacting devices to ensure stable performance [20, 39, 40]. Catechol groups are the key component of mussel foot proteins to exert adhesion. They bind tightly to many different surfaces, even in humid environments, through the formation of covalent bonds and noncovalent bonds [41–43]. Therefore, the catechol groups of C-PU-PTMEGs should also provide a wide range of adhesion properties besides the internal stabilization of the polymer. So, the adhesion of C-PU-PTMEGs was characterized to a variety of surfaces. Almost all C-PU-PTMEGs were removed from polytetrafluoroethylene (PTFE) and polyethylene (PE) surfaces (Figure S7) by a 3 M adhesive tape test, while the C-PU-PTMEG retentions were above 95% on all metallic materials (Figure 3(a)), revealing a strong adhesive effect on metallic materials. This was probably caused by the physical interaction of the catechol structure with metal ions and metal oxides via coordinate and hydrogen bonds [44] (Figure 3(b)), while the adhesion to hydrophobic polymers PE and PTFE was low. Further, a heavy weight of 32 kg could be suspended under two bonded 316L stainless steel (316L SS) sheets by a 40 *μ*m thick C-PU-PTMEG (1 K) film with a bonding area of 2 cm × 2 cm (Figure 3(c) and Video S1), showing a superb adhesion.

Standardized adhesion force evaluation using a universal tensile machine according to ASTM D1002 was performed (Figure 3(d)). The shear adhesion strength of C-PU-PTMEGs on metallic materials was generally higher than on PTFE and PE (Figure 3(e)), consistent with the previous findings. Even on different metallic materials, C-PU-PTMEGs exhibited different adhesion, which may be related to the characteristics of metal ions, such as their atomic weight, valence, oxidizability, and coordination form [45, 46]. What is more, the adhesive strength of C-PU-PTMEGs on the same material was positively correlated with the catechol content. Increasing molecular weight of the PTMEG fragment, associated with a decreased catechol structure content, allowed tuning of the binding ability of C-PU-PTMEGs. Due to the PUs served in a humid environment, C-PU-PTMEGs exhibited decreasing adhesions with immersing time up (Figure 3(f)), which was related to the oxidation of phenolic hydroxyl groups and the destruction of hydrogen bonds. However, C-PU-PTMEGs retained relatively strong shear adhesion after 14 days of immersion, even the worst-performing C-PU-PTMEG (2 K) owned a shear adhesion strength of 305 KPa, which was caused by that the hydrophobic PTMEG fragments dispersed the hydrated water on the interface and stabilized the underwater adhesion by enhancing the interaction between the adhesion groups and the substrates [47]. This behavior is critical for blood-contacting applications to ensure proper function as a coating.

### 2.3. Self-Healing Characteristics

**Figure 4:**
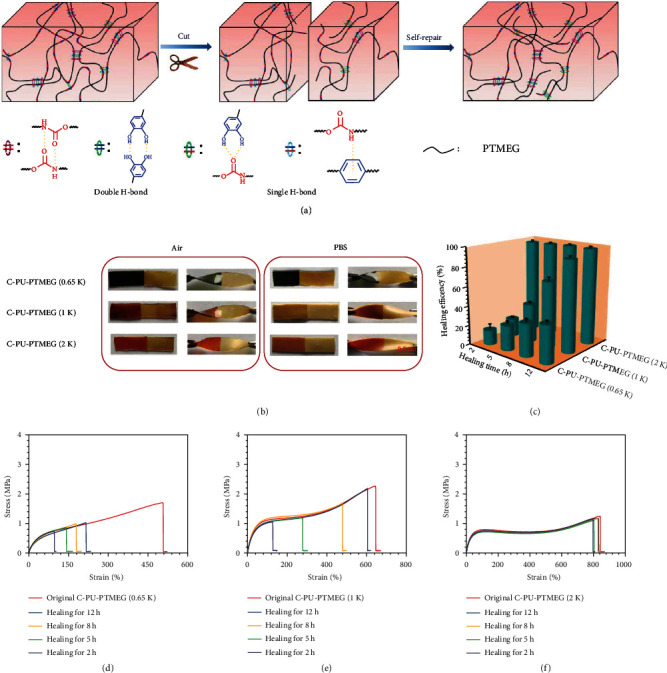
Self-healing of the C-PU-PTMEGs. (a) Schematic illustration of self-healing process through a variety of hydrogen bond interactions and a dynamics of molecular chain segments for C-PU-PTMEGs. (b) Digital photographs of C-PU-PTMEGs healing in air and PBS for 5 h at 37°C temperature after being cut into two pieces. (c) Dependency of self-healing efficiency of C-PU-PTMEGs on time at 37°C. Tensile stress-strain curve of the original and self-healing (d) C-PU-PTMEG (0.65 K), (e) C-PU-PTMEG (1 K), and (f) C-PU-PTMEG (2 K) after different healing times at a 37°C temperature in PBS.

Developing materials with self-repair in living organisms is of great application [48, 49], one of which is the possibility to extend its service life by functional recovery after damage by external actions. The mobility of the chain segments and the dynamic hydrogen bonds should endue C-PU-PTMEGs with self-healing properties (Figure 4(a)). For testing, a strip of C-PU-PTMEGs was cut into two pieces, which subsequently were gently brought in contact again. After one minute of contact at room temperature, C-PU-PTMEG (1 K) and C-PU-PTMEG (2 K) showed no fracture in the wound site after bending and twisting, while C-PU-PTMEG (0.65 K) exhibited partial rupture in the wound (Video S2, S3, S4). Subsequently, the strips were healed at 37°C for 5 h; all C-PU-PTMEG types could be healed in air and PBS (Figure 4(b)); however, C-PU-PTMEG (0.65 K) broke under 200 g weight (Figure S8).

In order to accurately describe the self-healing ability of the C-PU-PTMEGs in a similar *in vivo* environment, mechanical tests were performed when the C-PU-PTMEGs were healed in PBS for various periods of time at 37°C. C-PU-PTMEGs had hardly any swelling behavior in PBS solution, which ensured that all sample strips had the same size (Figure S9).  Figure 4(c) showed great differences in the self-healing capabilities of the different C-PU-PTMEG polymers. C-PU-PTMEG (2 K) had the highest self-repair efficiency, reaching 96.7% of the initial strength within only 2 h, which was almost complete repair (Figure 4(f)). For C-PU-PTMEG (1 K), the fracture stress and strain increased with the repair time, showing the time-dependency of the repair efficiency (Figure 4(e)). After 12 h of repairing time, the healing efficiency reached 92.8%. C-PU-PTMEG (0.65 K) presented the worst mechanical recovery (Figure 4(d)) with a repair efficiency of only 38.4% through 12 h. In addition, the mechanical properties (tensile strength and breaking elongation) of C-PU-PTMEGs decreased after PBS solution soaking (Figure S10A and B), which might be caused by changes in the cohesiveness of the polymer due to the partial breaking of the hydrogen bonds by the intervention of water [50]. These results confirmed that C-PU-PTMEGs had a self-healing ability that depends on the molecular weight of PTMEG fragments. On the one hand, the catechol structure of C-PU-PTMEGs increased the intermolecular exchangeable hydrogen bonds, which provided the driving force for self-repair. On the other hand, the various molecular weights of PTMEG caused different ordered chain segments, microphase separation, and Tg and thus brought about the diverse mobility of molecular chain segments. Therefore, the ability for self-healing could be tuned by adjusting the molecular weight of PTMEG fragment.

### 2.4. Stent Coating and *In Vitro* Cytocompatibility

**Figure 5:**
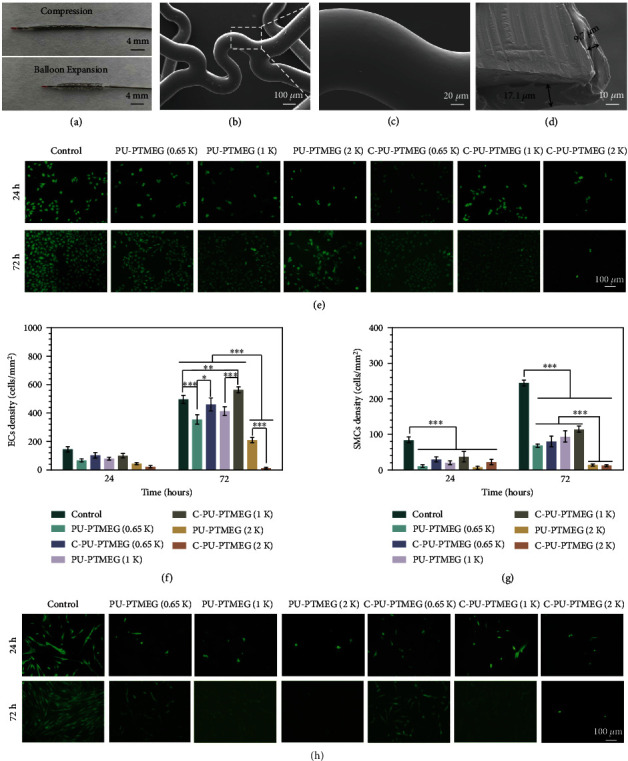
C-PU-PTMEG (1 K) as a coating of vascular stents and the cellular growth behavior of endothelial (HUVEC) and smooth muscle (HUASMC) cells on the various C-PU-PTMEG polymers. (a) Digital photograph of a vascular stent with C-PU-PTMEG (1 K) coating after compression and balloon expansion. (b) SEM morphology of the vascular stent with C-PU-PTMEG (1 K) coating after balloon expansion and (c) high magnification of the marked area. (d) Cross-sectional image of the stent with C-PU-PTMEG (1 K) coating. (e) Fluorescence images by cell tracker (CellTracker™ Green BODIPY®, Thermo Fisher Scientific, Inc.) and (f) proliferation of HUVECs on the PU surfaces. (g) Proliferation and (h) fluorescence images of HUASMCs on the PU surfaces. Statistical significance was regarded as follows: ^∗^*P* < 0.05,  ^∗∗^*P* < 0.01, and^∗∗∗^*P* < 0.001.

Due to the tunable multifunctionality, C-PU-PTMEG polymers are supposed to be used in a wide range of blood-contacting applications such as heart valves, vascular catheters, hemodialysis membranes, artificial blood vessels, and stent coatings [51, 52]. However, these applications are inevitably subject to forces such as bending or crushing. Therefore, the performance of C-PU-PTMEGs after application of forces was evaluated. Vascular stents, as typical blood-contacting devices that undergo both compression and expansion processes during handling, were selected to investigate the behavior of C-PU-PTMEG coatings upon forces. As stent coatings, C-PU-PTMEGs exhibited a smooth and flat surface without cracks and flaking (Figures 5(b) and 5(c) and Figure S11) after undergoing compression and balloon expansion for 1 min (Figure 5(a)); it demonstrated a tight adhesion to the substrate (Figure 5(d) and Figure S11). The C-PU-PTMEG coatings showed uneven thickness, with thicker coatings near the outside of stents in our coating process.

Subsequently, human umbilical vein endothelial cell (HUVEC) and human umbilical artery smooth muscle cell (HUASMC) cultures were performed on the different C-PU-PTMEG coatings to analyze the performance of the two main types of vascular cells on the polymers. Since 316L SS is widely used in blood environments, it was used as a control group for all biological characterizations in this study. As shown in Figures 5(e) and 5(f), PU-PTMEG (2 K) and C-PU-PTMEG (2 K) were cytotoxic, especially C-PU-PTMEG (2 K) with very few HUVECs observed after 72 hours of culturing. PU-PTMEGs with PTMEG segments of 0.65 and 1 kDa were basically able to maintain HUVEC growth. After catechol functionalization of the polymers, the growth of HUVECs was improved. Especially for C-PU-PTMEG (1 K), the proliferation of HUVECs was promoted compared to the control . It is known that an appropriate microphase separation structure favors cytocompatibility [53]. Here, PU-PTMEG (0.65 K), C-PU-PTMEG (0.65 K), PU-PTMEG (1 K), and C-PU-PTMEG (1 K) owned higher and approximate DPS, which might be the reason for the good HUVECs growth, while PU-PTMEG (2 K) and C-PU-PTMEG (2 K) exhibited the opposite behavior because of lower DPS. Subsequently, the results of culturing HUASMCs showed that the PU-PTEMGs and C-PU-PTEMGs significantly inhibited the proliferation of HUASMCs (Figures 5(g) and 5(h)).Especially, PU-PTMEG (2 K) and C-PU-PTMEG (2 K) strongly suppressed the HUASMC growth. Therefore, the C-PU-PTMEGs can be selected to promote, maintain, or inhibit the growth of HUVEC based on the selection of the PTMEG fragment, combined with the inhibition of SMC proliferation and other tunable properties, making it possible to realize customized blood-contacting devices.

### 2.5. Antioxidant and Anti-inflammatory Properties

Implanted materials generally cause an immunological response upon entry into the organism, leading to oxidative stress and inflammation [12], which in turn prevents tissue repair [13, 54] and causes neointimal hyperplasia or intravascular stenosis [21, 55]. This process is closely associated with inflammatory cells, especially macrophages and granulocytes [56]. Catechol is a natural antioxidant that quenches the excess reactive oxygen species (ROS)/reactive nitrogen species (RNS) produced at the site of inflammation, preventing tissue damage and inducing the production of anti-inflammatory mediators [33, 57, 58].

**Figure 6:**
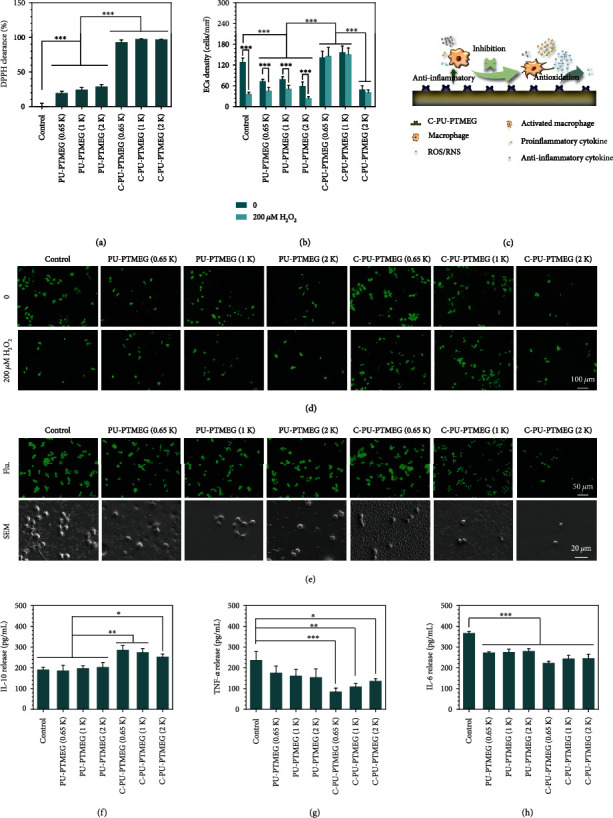
Antioxidant and anti-inflammatory evaluation. (a) The free radical clearing activity of the PU films by DPPH assay. (b) HUVECs proliferation on PU films in an oxidative stress condition mediated by 200 *μ*M H_2_O_2_ concentrations after culturing for one day. (c) Schematic presentation of the C-PU-PTMEGs for eliminating excess ROS/NOS and inhibiting inflammation and macrophage activation. (d) the fluorescent morphology of HUVECs by cell tracker on PU films in an oxidative stress condition mediated by 200 *μ*M H_2_O_2_ concentrations after culturing for one day. (e) Fluorescent and SEM images of macrophages culturing on the PU films after one day. Expression of anti-inflammatory factors (f) IL-10 and proinflammatory factors (g) TNF-*α* and (h) IL-6 in macrophages. Statistical significance was regarded as follows: ^∗^*P* < 0.05, ^∗∗^*P* < 0.01, and ^∗∗∗^*P* < 0.001.

It had to be checked whether the antioxidant and anti-inflammatory characteristics of catechol were successfully engrafted in the C-PU-PTMEG polymers. The antioxidant effect was probed by scavenging the 1,1-Diphenyl-2-picrylhydrazyl radical (DPPH). This was almost completed within only a 5 min period (Figure 6(a)), revealing a superb free radical scavenging ability. Subsequently, HUVECs were cultured in the presence of 200 *μ*M H_2_O_2_. After one day of culture, the number (Figure 6(b)) and morphology (Figure 6(d)) only on the three C-PU-PTMEGs were similar to those cultured without H_2_O_2_. These results demonstrated that C-PU-PTMEG polymers exhibit the antioxidant property of catechol for rapid scavenging of free radicals and prevent cellular damage from oxidative stress.

The anti-inflammatory effect of C-PU-PTMEGs was assessed using macrophages. As shown in Figure 6(e), macrophages on control and PU-PTMEGs were flattened and rounded with a few prominent structures, indicating that macrophages were activated. In contrast, macrophages on C-PU-PTMEGs showed a spherical shape without pseudopods and cellular degeneration, revealing an inhibited inflammatory state. Further, pro- and anti-inflammatory cytokines released by macrophages were measured. All three C-PU-PTMEG polymers enhanced the expression of anti-inflammatory IL-10 (Figure 6(f)) and suppressed the production of proinflammatory cytokines TNF-*α* and IL-6 (Figures 6(g) and 6(h)). Interestingly, the PTMEG fragment with low molecular weight seemed to enhance the expression of anti-inflammatory cytokines by macrophages on C-PU-PTMEGs as well as reduce proinflammatory cytokines, which may be attributed to the higher catechol content.

The *in vivo* inflammatory behavior of the C-PU-PTMEGs was additionally analyzed by subcutaneous implantation in SD rats (Figure S12). Here, the number of inflammatory cells and the thickness of the fibrous capsule around the C-PU-PTMEGs were significantly reduced during the acute inflammation period (2 weeks). As the implantation time reached 4 weeks, the fibrous capsule thickness on the surface of C-PU-PTMEGs increased, but the number of inflammatory cells and fibrous capsule thickness remained smaller than on the corresponding catechol-free PUs.

Thus, C-PU-PTMEGs modulated the expression of inflammatory factors and reduced the inflammatory response at the implantation site. The reason may be that catechol negatively regulates important transcription factors (NF-*κ*B) activation and nuclear translocation by eliminating oxidants and further regulating the expression of key cytokines and chemokines [59]. Interestingly, the capsule thicknesses of C-PU-PTMEG (0.65 K) and C-PU-PTMEG (1 K) were almost identical and much higher than that of C-PU-PTMEG (2 K) at different times, reflecting the tunable inflammation. Overall, C-PU-PTMEGs combined the antioxidant and anti-inflammatory properties of catechol (Figure 6(c)), and the inflammatory behavior can be controlled via modification of the PTMEG fragment.

### 2.6. Hemocompatibility

The performance of C-PU-PTMEGs in blood is a key property considering its application as a blood-contacting material. The adsorption of albumin, adhesion, and conformational changes of fibrinogen and as adsorption and activation of platelets are the key events in the blood response behavior [60].

**Figure 7:**
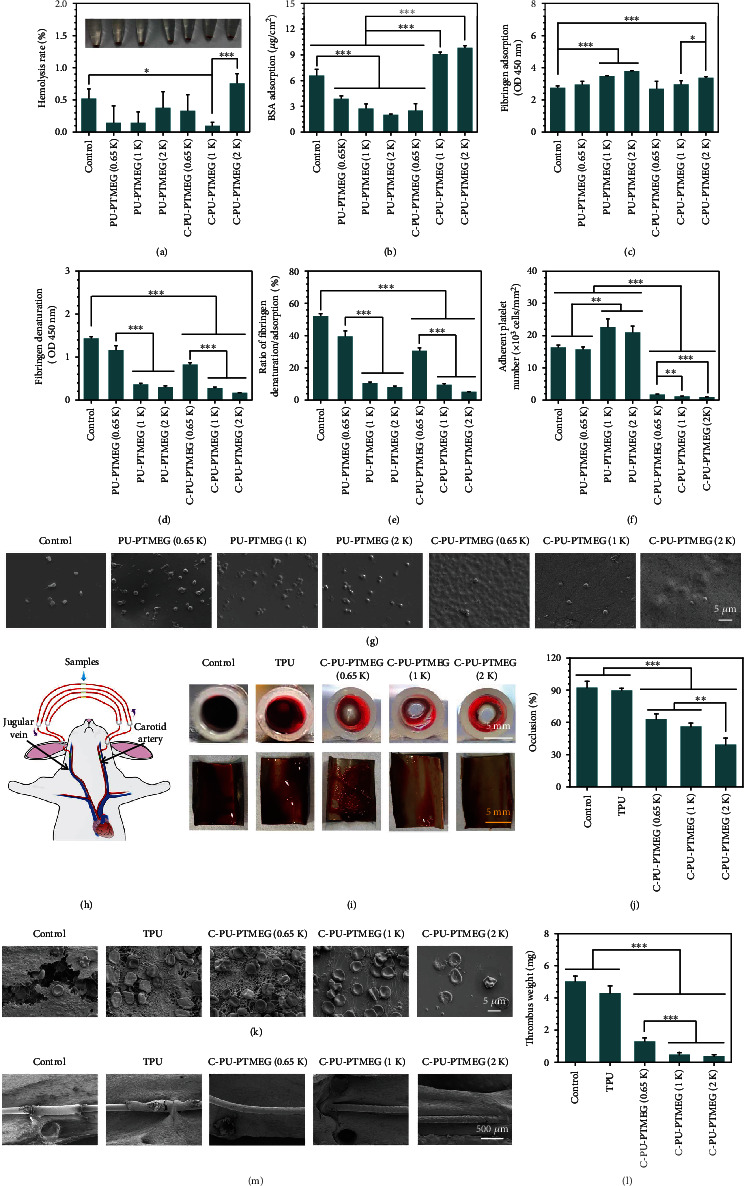
Hemocompatibility of C-PU-PTMEGs. (a) Hemolysis rate of various samples incubated by whole blood. (b) BSA adsorption on various films. (c) Fibrinogen adsorption and (d) denaturation on various films. (e) The ratio of the absorbance for fibrinogen denaturation and adsorption. (f) Platelet count and (g) the SEM image of various samples. Platelets from a healthy human volunteer platelet-rich plasma and incubated for 30 min on various samples. (h) Scheme of the ex vivo whole blood dynamic circulation system. (i) Cross-sectional photographs of the catheter and photographs of the sample after spreading to expose the thrombus. (j) Occlusion rate of the catheters containing various samples after one-hour circulation. (k) SEM images of the surface on samples going through the blood circulation. (l) Dry weight of the thrombi on the sample surface. (m) SME images of the samples in the rat abdominal aorta after implanting for 15 days. Statistical significance was regarded as follows: ^∗^*P* < 0.05,  ^∗∗^*P* < 0.01, and^∗∗∗^*P* < 0.001.

The hemolysis induced by C-PU-PTMEGs was within the safety level of 1%, far below the acceptable threshold of 5% (Figure 7(a)). As shown in Figure 7(b), C-PU-PTMEG (1 K) and C-PU-PTMEG (2 K) presented good bovine serum albumin (BSA) adsorption, while C-PU-PTMEG (0.65 K) exhibited low albumin affinity. Generally, albumin adsorption tends to be a thromboprotective property [61]. Interestingly, the higher molecular weight of PTMEG fragments would increase the fibrinogen adsorption of C-PU-PTMEGs (Figure 7(c)) and instead cut down the conformational changes of fibrinogen (Figure 7(d)). Moreover, the PUs showed a decrease in both fibrinogen adsorption and denaturation after the introduction of catechol. We made a ratio of fibrinogen denaturation/adsorption to illustrate the effect of the PUs on fibrinogen. As shown in Figure 7(e), the exhibiting result was similar to fibrinogen denaturation, suggesting that more fibrinogen was adsorbed as an increasing molecular weight of the PTMEG fragment, but only a fewer amount of fibrinogen was activated. Only the fibrin converted from activated fibrinogen promotes coagulation [62]. In addition, there were a reduced number of platelets adhering to the surface of the C-PU-PTMEGs (Figure 7(f)), and the images (Figure 7(g)) revealed low activation of the platelets with resting morphology on C-PU-PTMEGs (Figure S13).

These results confirmed the hematological safety of C-PU-PTMEGs and also indicated that the higher molecular weight of PTMEG fragment facilitated the coagulation protection of C-PU-PTMEGs. These static *in vitro* results were further validated in a dynamic ex vivo whole blood circulation study. Samples deposited on 316L SS foils were curled up and placed onto the inner walls of commercially available trigeminal blood tubes, which then connected to a rabbit arteriovenous vein (AV) as shown in Figure 7(h). After 1 h of circulation, the tube containing 316L SS as control and commercial PU (TPU, 2363-80A DOW®, USA) was almost clogged (Figure 7(i)), while the C-PU-PTMEGs remained open. Increased molecular weight of PTMEG was associated with lower occlusion rates (Figure 7(j)). After removing the samples and spreading them out (Figure 7(i)), the C-PU-PTMEG films had less thrombus covering the surface than control and TPU, and the thrombus mass was also significantly reduced (Figure 7(l)). Further SEM analysis of the samples revealed that both control and TPU surface were covered with typical thrombus (Figure 7(k)), showing extended fibrin networks and trapped red blood cells, and accompanied by echinocytes transformed from erythrocytes. For the C-PU-PTMEG surfaces, the C-PU-PTMEG (0.65 K) was less covered with fibrin network and erythrocytes than the TPU. Increasing the molecular weight of the PTEMG fragment to 1 kDa, only a few fibrins and erythrocytes were on the C-PU-PTMEG (1 K) surface. Almost no fibrin network and only a few adherent erythrocytes were discovered on the C-PU-PTMEG (2 K) sample.

The coagulation protection of C-PU-PTMEGs *in vivo* was further investigated by depositing it on 316L SS wires. All samples displayed no significant thrombotic deposition after implanting for 15 days in the rat abdominal aorta (Figure 7(m), Figure S14B), showing a good long-term *in vivo* hemocompatibility. Interestingly, the control and TPU surfaces were already covered with incomplete proliferative tissue, while little proliferative tissue was observed on the C-PU-PTMEGs, except for a small amount of protein adsorbed on the surface of the C-PU-PTMEGs (0.65 K) (Figure S14B). In addition, the surface of commercial TPU was concave (Figure S14A), which crumpled and aggregated during implantation due to low adhesion (Figure S14B), suggesting that C-PU-PTMEGs have better film formation and adhesion than commercial TPU.

These results indicated that C-PU-PTMEGs had less thrombogenic capacity than 316L SS and commercial TPU, and this thromboprotection increased with increasing molecular weight of the PTMEG fragment, which was consistent with the findings of *in vitro* studies. It did not stimulate thrombosis even during the 15 days of implantation *in vivo.* Overall, the blood behavior of C-PU-PTMEGs could be adjusted to a certain extent.

### 2.7. C-PU-PTMEGs with Tunable Multifunctionality

**Figure 8:**
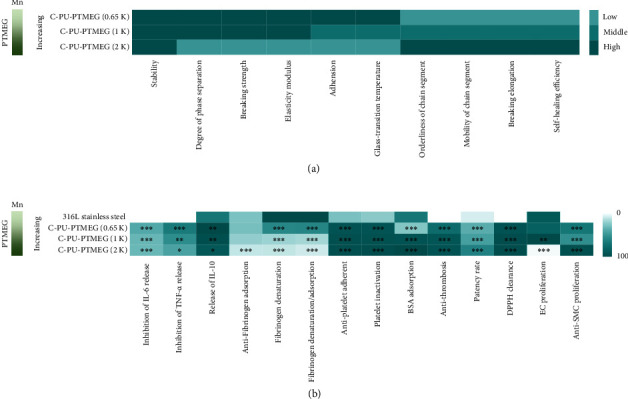
Heat maps of properties and functions for C-PU-PTMEGs. (a) Heat map of the physicochemical properties and function. (b) Heat map of C-PU-PTMEG-biological properties and function. Statistical significance was regarded as follows: ^∗^*P* < 0.05,  ^∗∗^*P* < 0.01, and^∗∗∗^*P* < 0.001.

To better reveal the relationship between the structure and the performance, the key results of C-PU-PTMEGs were summarized in heat maps by normalizing the properties (Figure 8). Firstly, we summarized the physicochemical properties of C-PU-PTMEGs (Figure 8(a)). Increasing molecular weights of PTMEG fragment caused regular changes in the structure and composition of C-PU-PTMEGs, such as reduced DPS, elastic modulus, adhesion, and Tg, and conversely increased the mobility of the chain segments, elongation at break and self-healing efficiency. The biological behavior and functions of C-PU-PTMEGs exhibited similar dependencies on the molecular weight of PTMEG (Figure 8(b)). Increased molecular weight of PTMEG fragment improved the antithrombotic capacity of C-PU-PTMEGs and slightly decreased anti-inflammatory activity. Also, the cellular behavior of endothelial and smooth muscle cells depended on the PTMEG molecular weight.

This allows selecting suitable property-specific applications. For example, the good mechanical properties, strong adhesion, antithrombogenicity, anti-inflammation, promotion of EC proliferation, and inhibition of SMC proliferation of C-PU-PTMEG (0.65 K) and C-PU-PTMEG (1 K) would meet the needs of artificial heart valves and stent coatings [4, 63–68]. Further increasing the molecular weight of PTMEG to obtain C-PU-PTMEG (2 K) had rapid self-healing efficiency, strong anticoagulation, inhibition of cell proliferation, and tissue proliferation, which had the potential to be applied to blood catheters and their coatings [69–73]. Therefore, the synthesized C-PU-PTMEGs could be tuned as needed to achieve the desired performance and multifunctionality for practical applications.

## 3. Conclusion

A series of catechol functionalized polyurethane-based elastomers (C-PU-PTMEGs) containing PTMEG as soft segment with superior metallic substrate adhesion, tunable self-healing efficiency, antioxidation, anticoagulation, anti-inflammatory properties, and cellular growth behavior were synthesized by catechol functionalization of PU-PTMEG elastomers. Here, the catechol played an important role in the integration of multiple functions through its own function and enriching hydrogen bonds of C-PU-PTMEGs. It was demonstrated that the variable PTMEG fragment affected the microstructure and catechol content of C-PU-PTMEGs, further resulting in the modulation of multiple properties and functions. These C-PU-PTMEG elastomers with controlled multiple functions and properties can be fit for the practical needs of complex blood applications and have a wide range of prospective usages in a variety of blood-contacting devices, such as artificial heart valves, cardiovascular stents, and catheters.

## 4. Materials and Methods

### 4.1. Materials

MDI, PTMEG (Mn:0.65, 1, 2 kDa), stannous octoate, sodium dodecyl sulfonate (SDS), fibrinogen, and bovine serum albumin (BSA) were purchased from Sigma-Aldrich. DMPA, 1-(3-Dimethylaminopropyl)-3-ethylcarbodiimide hydro (EDC), PBS powder, DPPH, calcium hydride, and 1-Hydroxybenzotriazole (HOBT) were purchased from Shanghai Aladdin Biochemical Technology Co., Ltd. DA and cell tracer were obtained from Thermo Fisher Scientific Inc. N,N-Dimethylformamide (DMF), Tetrahydrofuran (THF), N,N-Dimethylacetamide (DMAc), methanol, triethylamine (TEA), and hydrochloric acid (HCl) were supplied by Chengdu Chron Chemicals Co., Ltd. Except for DMAc, which used calcium hydride to remove the moisture, other reagents were used directly without further treatment.

### 4.2. Synthesis of PU-PTMEGs and C-PU-PTMEGs

PU-PTMEGs were gradually polymerized according to our previously reported methods in two steps: prepolymerization and chain extension [74]. The route and composition are shown in Figure 1(a) and Table S1, respectively. Firstly, PTMEG was stirred in vacuum at 80°C for 2 h before adding twice the molar amount of MDI. After two hours, 50 mL DMAc solution with DMPA and 0.1% stannous octoate were added into the two-neck flask under an argon atmosphere at 60°C for 6 h reaction. Then, the reaction solution was cooled to room temperature. Finally, PU-PTMEGs were obtained by precipitating in methanol and drying in vacuum for 72 h.

The synthesis of C-PU-PTMEGs was achieved by an amide reaction. Specifically, PU-PTMEGs were dissolved in DMF under an argon atmosphere. Then, 1.5 eq EDC and HOBT (for carboxyl) were added for reaction for 30 min in ice bath. 1 eq DA solution and 3.5 eq TEA were slowly dropped to react for 24 h at room temperature. Finally, C-PU-PTMEGs were precipitated in water (pH = 3) and dried in vacuum for 72 h.

### 4.3. Preparation of PU Films

The preparation of PU films was accomplished by solvent volatilization. The PUs (2 g) were dissolved in 10 mL THF and poured into a PTFE mold. After evaporation of the solvent, a series of different sizes of films was obtained according to the specific experimental requirements.

### 4.4. General Characterization

The PUs were measured by a Fourier Transform Infrared Spectrometer (FT-IR, NICOLET 5700, USA) in transmission condition at the range of 4000 to 500 cm^−1^, and the DPS of PUs was obtained after the analysis of infrared spectra (more details were provided in the Supplementary Information). The variable temperature FT-IR was carried out at a heating rate of 1°C min^−1^ in a temperature range of 25 to 120°C. The structure was further confirmed by ^1^H NMR (Bruker AV II-400).

The molecular weight was determined by gel permeation chromatography (GPC) using HLC-8320, which used THF as an eluent and polystyrene as a standard in the flow rate of 0.6 mL min^−1^ at 40°C.

Differential scanning calorimetry (DSC) was acquired by a differential scanning calorimeter (204F1D-0088-L). Eight mg of each sample was taken, sealed in an aluminum pan, and heated from room temperature to 120°C at a heating rate of 10°C min^−1^ under a N_2_ atmosphere, then, cooled to -80°C, and next reheated to 120°C at a heating rate of 5°C min^−1^. The second heating curves were acquired.

Thermogravimetric (TG) analysis was carried out by TGA (209F1E-0118-L). C-PU-PTMEGs (8 mg) were encapsulated in Al_2_O_3_ crucible and heated at a heating rate of 10°C min^−1^ in the range of 30 to 600°C under a N_2_ atmosphere.

The degree of crystallization of PU films was confirmed by XRD measurement (Empyrean) using CuK_*α*_ radiation generator (*λ* = 0.154 nm) at a scanning range from 10 to 90°.

The morphology of platelets and macrophages was observed by using scanning electron microscopy (SEM, JSM 7800F, Japan) in the presence of 15 nm gold layer at a 5 kV acceleration voltage.

Tensile tests were carried out using a uniaxial load test machine (SUNS, UTM4104X) with a crosshead speed of 200 mm min^−1^ at room temperature. The dumbbell-strips (length × width × thickness = 2 mm × 0.5 mm × 0.4 mm) were cut from large sheet films. The maximum stress on the curve was defined as the tensile stress, and the corresponding strain was regarded as the fracture strain. Elasticity modulus was calculated according to the slope of the linear region of the stress-strain curve. Each dumbbell-strip test was performed four times.

The dynamic mechanical analysis (DMA) of the samples (2 mm (*L*) × 0.5 mm (*W*) × 0.4 mm (*T*)) was investigated by TA Q800 (USA) at 1 Hz frequency at a heating rate of 3°C min^−1^ under a stretching mode, which was measured in the range of -100 to 100°C with an amplitude of 20 *μ*m.

### 4.5. Self-Healing Assay

The self-repair experiment was performed by cutting the sample strip into two parts and then immediately contacting the two parts for healing. The healing process was recorded by photography, bearing weight, and stretching. The stress-strain curves of the healed samples were obtained from the mechanical tests described above. The self-healing efficiency of the healed samples was calculated by the following equation:

(1) $$\begin{array}{c} \text{Healing effciency}\left(\%\right)=\times 100\%. \end{array}$$

### 4.6. Adhesion Test

The adhesive property of the PUs was evaluated by using cross-hatch adhesion and lap shear adhesion tests. More experimental details were provided in the Supplementary Information.

### 4.7. Spraying of Stent Coatings and 316L SS Wire

The preparation of vascular stent and 316L SS wire coatings was carried out by an ultrasonic atomizing applicator (Sono-Tek Corporation, USA). More experimental details were provided in the Supplementary Information.

### 4.8. In Vitro Cell Culture

Considering that the material implanted into the vasculature will inevitably interact with vascular cells, human umbilical vein endothelial cells (HUVECs) and human umbilical artery smooth muscle cells (HUASMAs) were used for the *in vitro* proliferation assessment evaluation of C-PU-PTMEGs. HUVECs were also used to evaluate the antioxidant effect. In addition, macrophages (RAW264.7) were used to evaluate the anti-inflammatory effects of C-PU-PTMEGs. More experimental details were provided in the Supplementary Information.

### 4.9. Antioxidant and Anti-inflammatory Tests

The antioxidant capacity of the material was assessed by DPPH scavenging and incubation of HUVECs in the presence of H_2_O_2_. The anti-inflammatory capacity of the material was evaluated by incubation of macrophages and subcutaneous implantation in SD rats. More experimental details are provided in the Supplementary Information.

### 4.10. Hemocompatibility Test

Here, the behavior of albumin, fibrinogen, and platelets on the PUs surface and the hemolysis were used to evaluate the hemocompatibility at steady condition. An ex vivo whole blood dynamic circulation system based on the New Zealand Great White rabbit was performed to assess the hemocompatibility of the PUs in a dynamic state. The samples were implanted into the rat abdominal aorta to investigated *in vivo* hemocompatibility. More experimental details are provided in the Supplementary Information.

### 4.11. Statistical Analysis

All quantitative assays in this study were performed with at least three independent samples. The data were analyzed in a one-way analysis of variance (ANOVA); date are presented as the mean ± standard deviation. The statistical significance was regarded as follows: ^∗^*P* < 0.05,  ^∗∗^*P* < 0.01, and^∗∗∗^*P* < 0.001.

## Data Availability

The datasets used and analyzed in this work can be available from the authors on reasonable request.

## Supplementary Materials

Supplementary Materials Table S1: composition and nomenclature of the PUs. Figure S1: ^1^H NMR spectra of (A) PU-PTMEGs and (B) C-PU-PTMEGs. Deuterium DMSO was used as the solvent of PU-PTMEG (0.65 K), C-PU-PTMEG (0.65 K), C-PU-PTMEG (1 K), and C-PU-PTMEG (2 K). Deuterium chloroform was used as the solvent of PU-PTMEG (1 K) and PU-PTMEG (2 K). (C) ^1^H NMR spectra and integrals of C-PU-PTMEGs between 6.2 and 7.5 ppm. Figure S2: FT-IR spectra of PU-PTMEG. Table S2: GPC results and degree of microphase separation of PU-PTMEG. Table S3: GPC results, catechol content, and degree of microphase separation of C-PU-PTMEGs. Figure S4: changes of the FI-TR spectrum of C-PU-PTMEGs with an increasing temperature in the range of 1150 to 1350 cm^−1^ and 3200 to 3550 cm^−1^, respectively. Figure S3: FT-IR spectra of C=O absorbance of PUs in the region of 1680-1770 cm^−1^. Detailed description of peaks is supplied in Table S4. Table S4: carbonyl group assignments and band percentage areas of the PUs in FT-IR. Figure S5: (A) XRD spectra of the PU-PTMEG. (B) Tan*δ* curves of the C-PU-PTMEGs in DMA with a range of -90 to 90°C temperature. Figure S6: (A) energy loss and (B) change of elastic modulus of C-PU-PTMEGs in successive loading-unloading cycles at a tensile rate of 100 mm min^−1^. Figure S7: photos of the cross-hatch tests after 5 min of 3 M tape treatment. Video S1: the joint bonded two pieces of 316L SS with a C-PU-PTMEG (1 K) film for one day can withstand a weight of 32 kg. Video S2: video of C-PU-PTMEG (0.65 K) healing in air for one minute after being cut into two pieces. Video S3: video of C-PU-PTMEG (1 K) healing in air for one minute after being cut into two pieces. Video S4: video of C-PU-PTMEG (2 K) healing in air for one minute after being cut into two pieces. Figure S8: a 200 g weight-bearing test was performed when repairing for 5 h at 37°C. Figure S9: the swelling rate of C-PU-PTMEGs in PBS solution at 37°C for different time. Figure S10: comparison of (A) tensile strength and (B) breaking elongation of C-PU-PTMEGs in dry and wet state. Statistical significance was regarded as follows: ^∗∗∗^*P* < 0.001. Figure S11: SEM morphology of the vascular stent with C-PU-PTMEG (0.65 K) and C-PU-PTMEG (2 K) coating after balloon expansion and SEM morphology of the local enlargement in the stent. Figure S12: histological and fibrous capsule thickness analysis of inflammatory response within the PUs films by H&E-stain. ∗ denotes the implantation location of the polymer films. The red double arrow represents the thickness of the new fibrous capsule. Figure S13: activation of platelet on various samples. Statistical significance was regarded as follows: ^∗∗∗^*P* < 0.001. Figure S14: SEM images of samples (A) before implantation and (B) after implantation in rat abdominal aorta for 15 days.
