# Supplementary material for: Adhesive and Self-Healing Polyurethanes with Tunable Multifunctionality
Source: Research (Wash D C). 2022 Oct 26;2022:9795682. doi: 10.34133/2022/9795682 (PMC9639449; doi:10.34133/2022/9795682)
Supplement: Supplementary Materials — Table S1: composition and nomenclature of the PUs. Figure S1: 1H NMR spectra of (A) PU-PTMEGs and (B) C-PU-PTMEGs. Deuterium DMSO was used as the solvent of PU-PTMEG (0.65 K), C-PU-PTMEG (0.65 K), C-PU-PTMEG (1 K), and C-PU-PTMEG (2 K). Deuterium chloroform was used as the solvent of PU-PTMEG (1 K) and PU-PTMEG (2 K). (C) 1H NMR spectra and integrals of C-PU-PTMEGs between 6.2 and 7.5 ppm. Figure S2: FT-IR spectra of PU-PTMEG. Table S2: GPC results and degree of microphase separation of PU-PTMEG. Table S3: GPC results, catechol content, and degree of microphase separation of C-PU-PTMEGs. Figure S4: changes of the FI-TR spectrum of C-PU-PTMEGs with an increasing temperature in the range of 1150 to 1350 cm−1 and 3200 to 3550 cm−1, respectively. Figure S3: FT-IR spectra of C=O absorbance of PUs in the region of 1680-1770 cm−1. Detailed description of peaks is supplied in Table S4. Table S4: carbonyl group assignments and band percentage areas of the PUs in FT-IR. Figure S5: (A) XRD spectra of the PU-PTMEG. (B) Tanδ curves of the C-PU-PTMEGs in DMA with a range of -90 to 90°C temperature. Figure S6: (A) energy loss and (B) change of elastic modulus of C-PU-PTMEGs in successive loading-unloading cycles at a tensile rate of 100 mm min−1. Figure S7: photos of the cross-hatch tests after 5 min of 3 M tape treatment. Video S1: the joint bonded two pieces of 316L SS with a C-PU-PTMEG (1 K) film for one day can withstand a weight of 32 kg. Video S2: video of C-PU-PTMEG (0.65 K) healing in air for one minute after being cut into two pieces. Video S3: video of C-PU-PTMEG (1 K) healing in air for one minute after being cut into two pieces. Video S4: video of C-PU-PTMEG (2 K) healing in air for one minute after being cut into two pieces. Figure S8: a 200 g weight-bearing test was performed when repairing for 5 h at 37°C. Figure S9: the swelling rate of C-PU-PTMEGs in PBS solution at 37°C for different time. Figure S10: comparison of (A) tensile strength and (B) breaking elongation [file 9795682.f1.zip › Supplementary Information (Clean Version).docx]

**Supplementary Information**

**Adhesive and Self-Healing Polyurethanes with** **Tunable Multifunctionality**

Lei Zhou^1^, Lu Zhang^1^, Peichuang Li^3^, Manfred F. Maitz^1,5^, Kebing Wang^1^, Tengda Shang^1^, Sheng Dai^1^, Yudie Fu^1^, Yuancong Zhao^1^, Zhilu Yang^2,4,^*, Jin Wang^1,^* and Xin Li^1,^*

^1^School of Materials Science and Engineering, Southwest Jiaotong University, Department of Cardiology, Third People’s Hospital of Chengdu Affiliated to Southwest Jiaotong University, Chengdu, 610031, Sichuan, China.

^2^Affiliated Dongguan Hospital, Southern Medical University, Dongguan, Guangdong, 523059, China

^3^Heze Branch, Qilu University of Technology (Shandong Academy of Sciences), Biological Engineering Technology Innovation Center of Shandong Province, Heze, 274000, China

^4^Guangdong Provincial Key Laboratory of Cardiac Function and Microcirculation, Guangzhou, Guangdong, 510080, China

^5^Max Bergmann Center of Biomaterials Dresden, Leibniz Institute of Polymer Research Dresden, Hohe Strasse 6, 01069 Dresden, Germany

*Correspondence should be addressed to Jin Wang; wangjin@swjtu.edu.cn, Xin Li; lixin131715@163.com and Zhilu Yang; [zhiluyang1029@smu.edu.cn](mailto:zhiluyang1029@smu.edu.cn), Lin Cai; cailinwm@163.com

1. **Experimental section**
   1. **Calculation of the** **degree of phase separation (DPS)**

Infrared spectroscopy is a common method to calculate the DPS in block PUs [1, 2]. The cornerstone is derived from the assumption that the hydrogen-bond components appear in the hard segment area, while the unbound groups consist in the soft segment. The DPS of PU is described by the intensity of the absorbance of the hydrogen-bond groups. Here, the carbonyl peak was selected to carry out deconvolution analysis in the region of 1680-1770 cm^-1^ by using PeakFit v4.12 software. Then the DPS was obtained by the following equation (1):

$DPS(\%)={A_{b}}/{A_{t}}$ (1)

Where *A_b_* and *A_t_* represented the absorbance values of hydrogen-bond groups and the sum of all groups, respectively.

Especially since the carbonyl from the amide part was not in this selected region and the high grafting rate of DA, an assumption that the carbonyl group in this region was all from the carbamate group proposed for C-PU-PTMEGs. However, the region of PUs contained the two carbonyls, one was from carboxyl, and the other belonged to carbamate. Hydrogen bonds formed by the carboxyl and carbamate were analyzed together (Table S1).

- 1. **Swelling behavior**

The swelling behavior of PU films was evaluated according to ASTM D570-98 [3]. Before immersion in phosphate-buffered saline (PBS, pH 7.4) at 37 °C, the weight of each dry sample (1×1 cm^2^) was recorded. At the present time points, the PU films were removed, and the residual water on the surface was adsorbed with a filter paper. The weigh of samples was recorded. The swelling ratio was calculated according to the following equation (2):

$Swelling ratio(\%)=(W_{w}-W_{d})/W_{d} \times100\%$ (2)

Where *W_w_* and *W_d_* represent the weights of the wet and dried PU films, respectively.

- 1. **Adhesion test**

The cross-hatch adhesion was measured using a special exact cutter in accordance with the requirements of ASTM D3359 [4]. Specifically, a 2 mm standardized cutting knife was used to obtain a square cut pattern in the target coating area. The sample was brushed using a magnifying glass (2.5x). Then the 3M tape was deposited on the pattern at a 45° angle and removed after 5 min. Adhesion strength was confirmed based on the percentage of residual coating area after tape peeling.

The lap shear adhesion test was performed in accordance with ASTM D1002 [5]. The bonding ability of the polymer to various substrates with a bonding area of 25 mm × 25 mm was tested using a uniaxial load test machine with a deformation speed of 1 mm min^-1^ at room temperature. The shear adhesion strength of the materials was obtained based on the ratio of the maximum load to the initial bonded area. The measurements were repeated three times for each sample.

- 1. **Spraying of stent coatings**

A cleaned 316L SS stent was placed on ultrasonic atomising applicator (Sono. Tek Corporation) and sprayed with 1% C-PU-PTMEGs solution at an atomisation power of 1.6 W and a feed flow rate of 0.1 ml min^-1^. The spraying was cycled 40 times at a horizontal rate of 0.25 cm s^-1^ with a rotate speed of 120 r min^-1^. After spraying and a complete evaporation of the tetrahydrofuran, the stents were removed and dried under vacuum for 24 h.

A cleaned 316L SS wire was placed on ultrasonic atomising applicator and sprayed with 1% polymers solution at an atomisation power of 1.6 W and a feed flow rate of 0.08 ml min^-1^. The spraying was cycled 20 times at a horizontal rate of 0.25 cm s^-1^ with a rotate speed of 120 r min^-1^. After spraying and a complete evaporation of the tetrahydrofuran, the wires were removed and dried under vacuum for 24 h.

- 1. ***In vitro*** **cells culture and fluorescent stain**

HUVECs were supported by Wuhan Service Biotechnology Co., Ltd. DME/F-12 medium (Hyclone) containing 10% fetal bovine serum (FBS, Hyclone) was used to culture ECs. After sterilizing the PU films, ECs with a density of 1.0 × 10^4^ cells/sample were seeded and cultured in 5% CO_2_ atmosphere at 37 ℃ for 1 and 3 days, respectively.

HUASMCs were also purchased from Wuhan Service Biotechnology Co., Ltd. And HUASMCs were cultured in SMCs culture medium (SMCM, Hyclone) containing 10% fetal bovine serum. HUASMCs were seeded on sterile PU films at a density of 1.0 × 10^4^ cells/sample and cultured in 5% CO_2_ atmosphere at 37 ℃ for 1 and 3 days, respectively.

For the H_2_O_2_-induced apoptosis test of ECs, 200 µM H_2_O_2_ was selected to evaluate the viability of ECs on the PU films [6]. The HUVECs were inoculated on the PU films at the density of 1.0 × 10^4^ cells/sample in the presence of H_2_O_2_ (final concentration 200 µM) at 37 ℃ under 5% CO_2_ atmosphere for one day.

The macrophages (RAW 246.7) were treated with a DMEM/HIGH glucose medium (Hyclone) containing 10% FBS. The macrophages were seeded at a density of 6×10^4^ cells/sample on sterile PU films and cultured in 5% CO_2_ atmosphere at 37 ℃ for one day. After that, 100 µL of the culture supernatant was taken for the detection of inflammatory factors. The operation procedures were the same as the instructions of ELISA kit (Wuhan ColorfulGene biological technology Co., Ltd,. China).

Cell tracer (CellTracker™ Green BODIPY®, Thermo Fisher Scientific, Inc.) was used to stain the cells in order to acquire the green fluorescence image of cell morphology and the number of cells. Specifically, the living cells on the samples were cleaned by stroke-physiological saline solution (SPSS) for 3 times to get rid of medium. After that, cell tracer was added and incubated at 37 °C for 30 min, and then PBS was used to remove the excess cell tracer. After fixing the cells with 4% paraformaldehyde supplied by Boster for 15 min, the samples were rinsed by PBS. An inverted fluorescence microscope (Olympus, IX51) was used to obtain a fluorescence image of the adhered cells, and the cell density was counted from 9 images using the software Image J.

- 1. **Intrinsic antioxidant activity**

The antioxidant activity of polymer films was evaluated via DPPH assay. Specifically, the PUs were dissolved in THF, and films were formed on a 24-well PTFE pore plate by solvent evaporation. At 37 ℃, the PU films were immersed in DPPH solution (0.1 mmol/L) in methanol. After 5 min, the absorbance value at 517 nm was measured. The antioxidant activity was calculated according to the following equation (3):

$Antioxidant ability(\%)=(A_{0}-A_{S})/A_{0}\times100\%$ (3)

Where *A_0_* and *A_S_* represented the absorbance values at 517 nm for the control and PUs, respectively.

- 1. ***In vivo* inflammatory response**

The subcutaneous inflammatory response of polymers was investigated using healthy male rats (Sprague Dawley, SD). All operation procedures were conducted in accordance with the Chinese Council of Guidelines for the Care and Use of Laboratory Animals. The sterilized polymer films were embedded under the back skin of SD rats and reared for 2 weeks and 4 weeks, respectively.

The tissue around the sample was then carefully removed and immersed in 4% paraformaldehyde for 1 day. After fixation, the tissue was cleaned in PBS, then ethanol was used for gradient dehydration. Then, the tissue was treated infiltrated with xylene and encapsulated in paraffin wax. Furthermore, Hematoxylin-eosin (H&E) was used for staining the tissue after cutting the paraffin blocks. Finally, the sections were observed and recorded via an optical microscope.

- 1. **Hemocompatibility test**

**1.8.1. BSA adsorption test**

A micro-BCA protein assay kit (Thermo Fisher Scientific, Inc., America) was used to determine the quantity of BSA adsorbed on the sample surface. Firstly, samples were soaked overnight in PBS. After that, 500 µL BSA solution (42 mg/mL) was added to the surface of the samples and incubated at 37 ℃ for 3 h. The samples were rinsed with PBS to move away unbound BSA. Then, the samples were immersed in SDS solution (1 mg/mL) for 24 h. The absorbance of each sample at 562 nm was measured using the kit, and the quantity of BSA adsorption was calculated according to the standard.

**1.8.2. Fibrinogen adsorption and denaturation tests**

Enzyme-linked immunosorbent assay (ELISA) was used to investigate the behavior of fibrinogen. Specifically, 500 µL fibrinogen solution (2 mg/mL) in the presence of 3 wt% BAS was taken on the surface of the samples and incubated at 37 ℃ for 2 h. Then, the unbound fibrinogen was removed with PBS. After that, the fibrinogen was blocked by immersing in 1 wt% BSA solution for 30 min. Later, 25 µL horseradish peroxidase (HRP)-labeled goat anti-human fibrinogen antibody (1/100, bs-1240G-HRP, Biss) solution was added to cover the surface of samples and incubated at 37 ℃ for 1 h after rinsing with PBS. Furthermore, 3,3,5,5-tetramethylbenzidine (TMB) solution was covered to the surface to form chromogen for 8 min. Subsequently, 1 wt% sulfuric acid was added to terminate the reaction. Finally, the adsorption result of fibrinogen was obtained by detecting the absorbance of the reaction solution at 450 nm. The previous antibody was replaced by rabbit anti-human fibrinogen gamma chain antibody (1/500 Dilution in PBS, Product No: BS1240G, Bioss) and goat anti-rabbit IgG/HRP antibody (1/1000 Dilution in PBS, Product No.:Bs - 0295 - g - HRP, Bioss). The results of fibrinogen degeneration were obtained.

**1.8.3. Hemolysis rate test**

Firstly, 4 mL whole blood was diluted with 5 mL stroke-physiological saline solution (SPSS). Meanwhile, samples were immersed in 9.8 mL SPSS at 37 ℃ for 30 min. All samples were added with 0.2 mL diluted whole blood and incubated at 37 ℃ for 1 h. After that, the samples were removed and centrifuged at 3000 rpm for 5 min. The absorbance of the supernatant at 540 nm was measured by a microplate reader (EPOCH 2, America). Then the hemolysis rate was calculated by the following equation (4):

$Hemolysis rate(\%)=(A_{S}-A_{N})/(A_{P}-A_{N} )\times100\%$ (4)

Where *A_S_*, *A_N_* and *A_P_* represented the absorbance values of PUs, negative and positive controls, respectively.

**1.8.4. Platelet adhesion and activation test**

Platelet-rich plasma (PRP) was obtained from fresh whole blood, collected from healthy volunteers, by centrifugation at 1500 rpm. Immediately, 70 µL PRP was added to the surface of each sample and incubated at 37 ℃ for 1 h. Then, the sample surfaces were washed three times with PBS to remove unbound platelets and fixed overnight with 2.5% glutaraldehyde. Two groups were separated, one of which was stained with rhodamine and the other group was observed by SEM after dehydration, dealcoholization and critical point drying.

**1.8.5. *Ex vivo* dynamic circulation of the whole blood**

An *ex vivo* whole blood dynamic circulation system based on New Zealand White Rabbit was established to assess the hemocompatibility of the polymer in a dynamic state. All operations were in accordance with the Local Ethical Committee and Laboratory Animal Administration Rules of China. The system consisted of tubes with the samples inserted, connected to the carotid artery and jugular vein of a rabbit. Prior to the start of the experiment, a polymer coating deposited on 316L SS foil was placed in the pipe. The foil was removed after 1 hour of blood circulation and rinsed 3 times with saline. Then, the surfaces of the samples were recorded with a digital camera. After fixing with glutaraldehyde aqueous solution (2.5%) for one day, the samples gone through dehydrating, de-alcoholizing and platting gold. Lastly, a SEM was used to observe the morphology of the coating.

**1.8.6. *In vivo* evaluation of hemocompatibility**

All polymers were deposited on 316L SS wire (φ = 0.1 mm). Male Sprague Dawley (SD) rats were subjected to general anesthesia and anticoagulation, and the abdominal aorta was isolated. The samples were subsequently implanted, fixed and the wounds were sutured. All operations were in accordance with the Local Ethical Committee and Laboratory Animal Administration Rules of China. 15 days after implantation, the abdominal aorta containing the sample was removed from SDs and fixed with 4% paraformaldehyde for 3 days. The arterial vassel was dissected and it was observed by SEM after dehydration and decolonization.

**2. Results section**

**2.1. The composition of the PUs**

**Table S1.** The composition and nomenclature of the PUs

| **Sample** | **PTMEG**  **(Mn:0.65 kDa)** | **PTMEG**  **(Mn:1 kDa)** | **PTMEG**  **(Mn:2 kDa)** | **MDI** | **DMPA** | **DA** | **Hard segment (wt%)** |
| --- | --- | --- | --- | --- | --- | --- | --- |
| PU-PTMEG (0.65K) | 1 | / | / | 2 | 1 | / | 49.4 |
| PU-PTMEG (1K) | 1 | / | / | 2 | 1 | 1 | 54.2 |
| PU-PTMEG (2K) | / | 1 | / | 2 | 1 | / | 38.8 |
| C-PU-PTMEG (0.65K) | / | 1 | / | 2 | 1 | 1 | 43.5 |
| C-PU-PTMEG (1K) | / | / | 1 | 2 | 1 | / | 24.1 |
| C-PU-PTMEG (2K) | / | / | 1 | 2 | 1 | 1 | 27.8 |

Unit: mol, / stands for no inputs

**2.2. ^1^H NMR**

**
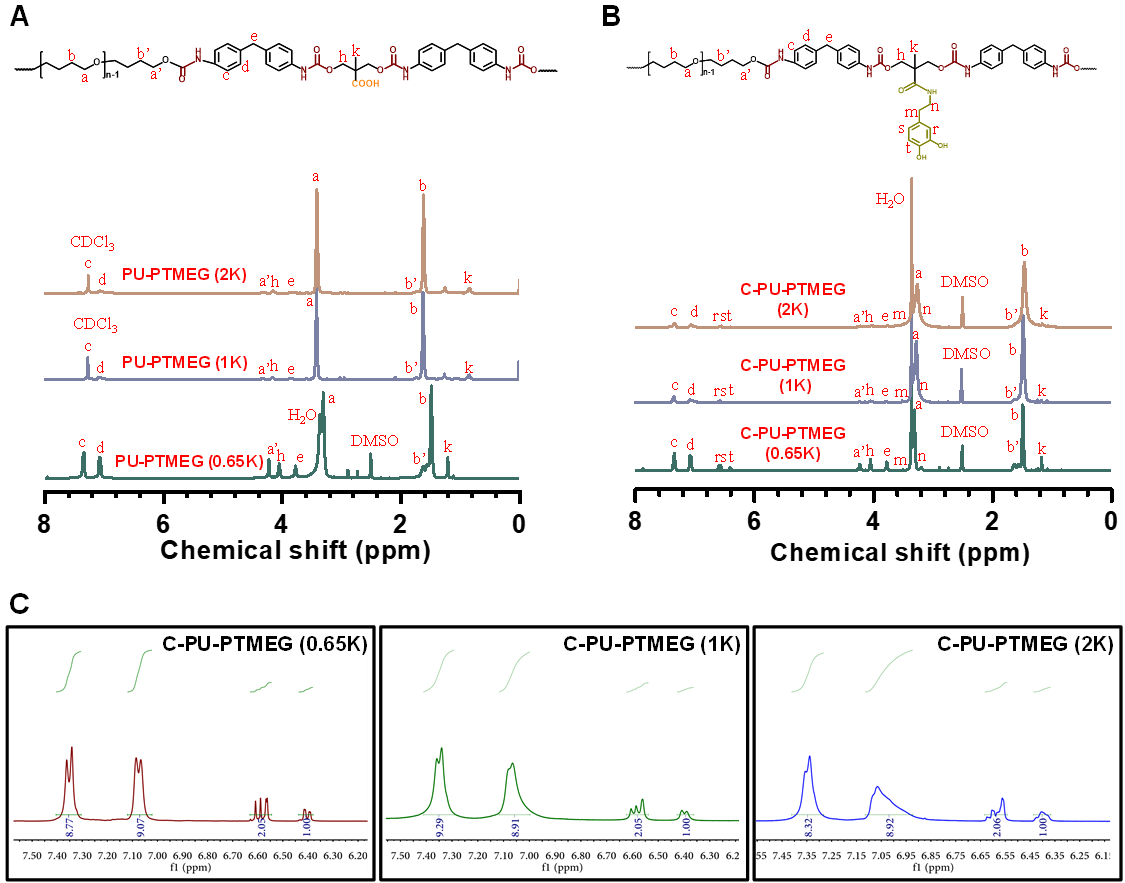
**

**Fig. S1.** ^1^H NMR spectra of (A) PU-PTMEGs and (B) C-PU-PTMEGs. Deuterium DMSO was used as the solvent of PU-PTMEG (0.65K), C-PU-PTMEG (0.65K), C-PU-PTMEG (1K) and C-PU-PTMEG (2K). Deuterium chloroform was used as the solvent of PU-PTMEG (1K) and PU-PTMEG (2K). (C) ^1^H NMR spectra and integrals of C-PU-PTMEGs between 6.2 and 7.5 ppm.

**2.3. GPC, catechol content and DPS**

**Table S2.** GPC results and degree of microphase separation of PU-PTMEGs.

| **Sample** | **Mn (kDa)** | **Mw (kDa)** | **PDI** | **DPS (%)** |
| --- | --- | --- | --- | --- |
| PU-PTMEG (0.65K) | 33.2 | 80.0 | 2.4 | 63.4 |
| PU-PTMEG (1K) | 24.7 | 41.9 | 1.7 | 57.7 |
| PU-PTMEG (2K) | 49.4 | 121.1 | 2.4 | 40.9 |

**Table S3.** GPC results, catechol content and degree of microphase separation of C-PU-PTMEGs.

| **Sample** | **Mn (kDa)** | **Mw (kDa)** | **PDI** | **catechol grafting rate (%)** | **catechol content**  **(wt %)** | **DPS (%)** |
| --- | --- | --- | --- | --- | --- | --- |
| C-PU-PTMEG (0.65K) | 35.2 | 142.4 | 4.071 | 94.3 | 10.2 | 63.8 |
| C-PU-PTMEG (1K) | 27.2 | 50.5 | 1.856 | 91.8 | 7.9 | 62.2 |
| C-PU-PTMEG (2K) | 55.1 | 142.8 | 2.591 | 88.3 | 4.9 | 51.7 |

**2.4. FT-IR**

**
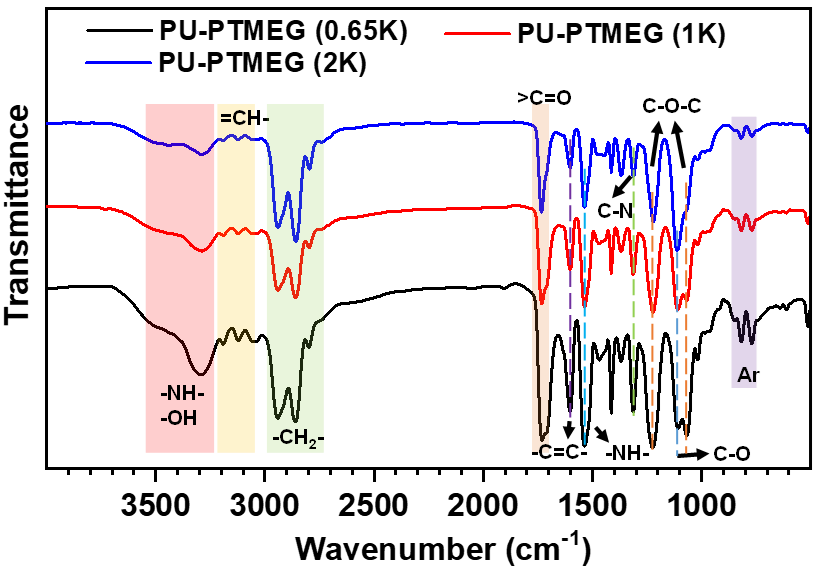
**

**Fig. S2.** FT-IR spectra of PU-PTMEGs.

**2.5. Gauss model fitting**

**
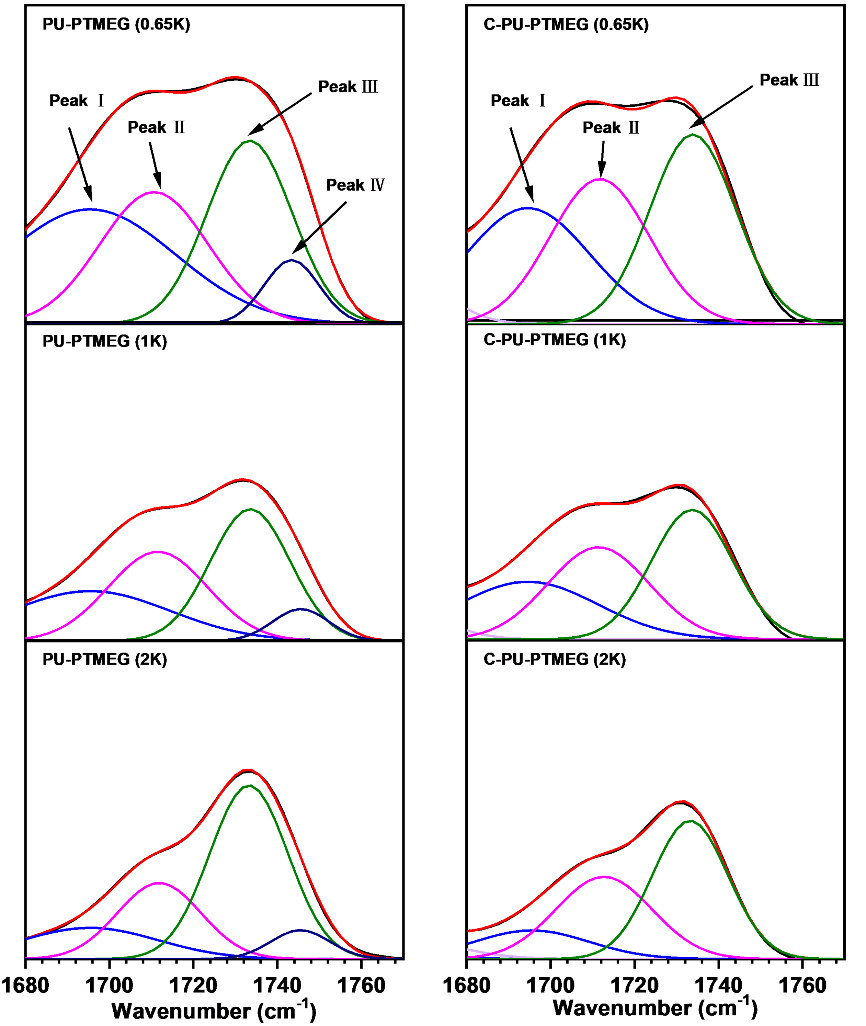
**

**Fig. S3.** FT-IR spectra of C=O absorbance of the PUs in the region of 1680-1770 cm^-1^. Detailed description of peaks is supplied in Table S4.

**Table S4.** Carbonyl group assignments and bands percentage areas of the PUs in FT-IR.

| **Sample** | **>C=O band** | | | | | | | |
| --- | --- | --- | --- | --- | --- | --- | --- | --- |
|  | **Peak Ⅰ:H-bond in soft segment** | | **Peak Ⅱ:H-bond in hard segment** | | **Peak Ⅲ:free C=O in**  **–NHCOO-** | | **Peak Ⅵ:free C=O in**  **-COOH** | |
|  | **Wavenumber (cm^-1^)** | **Proportion (%)** | **Wavenumber (cm^-1^)** | **Proportion (%)** | **Wavenumber (cm^-1^)** | **Proportion (%)** | **Wavenumber (cm^-1^)** | **Proportion (%)** |
| PU-PTMEG (0.65K) | 1695.4 | 36.5 | 1710.6 | 26.9 | 1733.4 | 29.7 | 1745.3 | 6.9 |
| PU-PTMEG (1K) | 1695.3 | 27.4 | 1711.5 | 30.3 | 1733.5 | 36.2 | 1745.5 | 6.1 |
| PU-PTMEG (2K) | 1695.4 | 16.4 | 1711.8 | 24.5 | 1733.2 | 52.6 | 1745.4 | 6.5 |
| C-PU-PTMEG (0.65K) | 1694.5 | 31.8 | 1711.7 | 32.0 | 1733.8 | 36.2 | \ | \ |
| C-PU-PTMEG (1K) | 1694.5 | 28.9 | 1711.4 | 33.3 | 1733.6 | 37.8 | \ | \ |
| C-PU-PTMEG (2K) | 1695.5 | 15.2 | 1712.6 | 36.5 | 1733.2 | 48.3 | \ | \ |

**2.6. The variable-temperature FTIR**


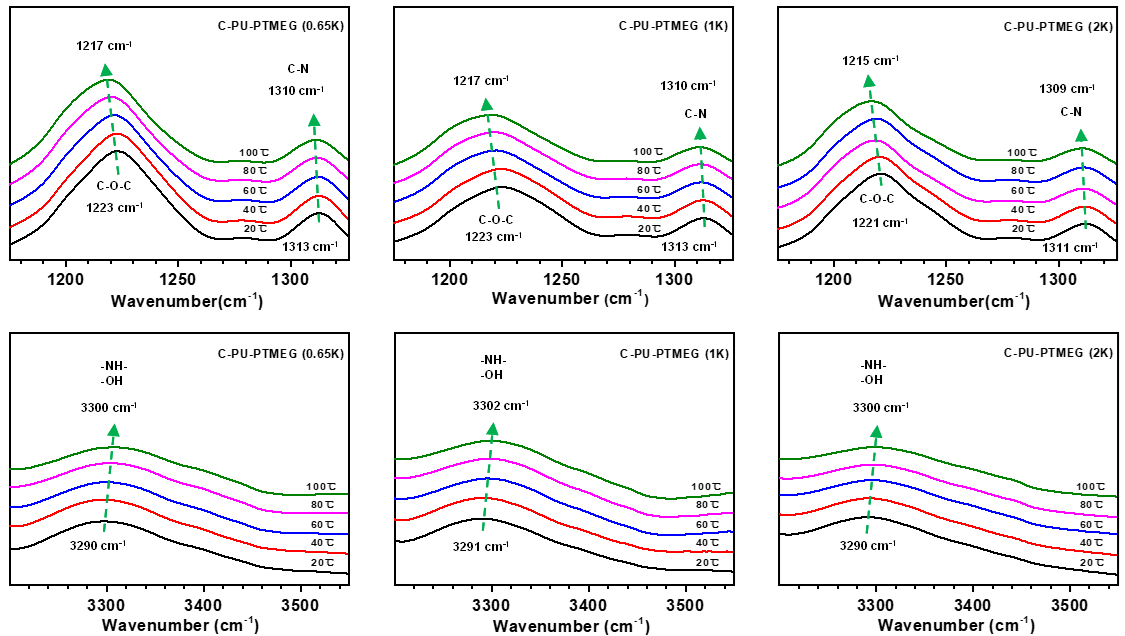


**Fig. S4.** Changes of FI-TR spectra of C-PU-PTMEGs with an increasing temperature in the range of 1150 to 1350 cm^-1^ and 3200 to 3550 cm^-1^, respectively.

**2.7. XRD and DMA**


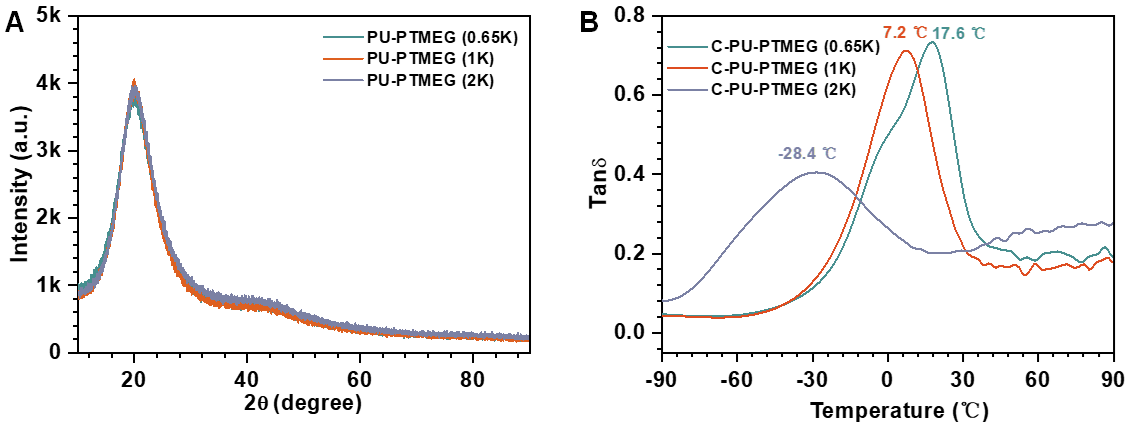


**Fig. S5.** (A) XRD spectra of the PU-PTMEGs. (B) Tanδ curves of the C-PU-PTMEGs in DMA with a range of -90 to 90 ℃ temperature.

**2.8.** **Cyclic tensile test**

**
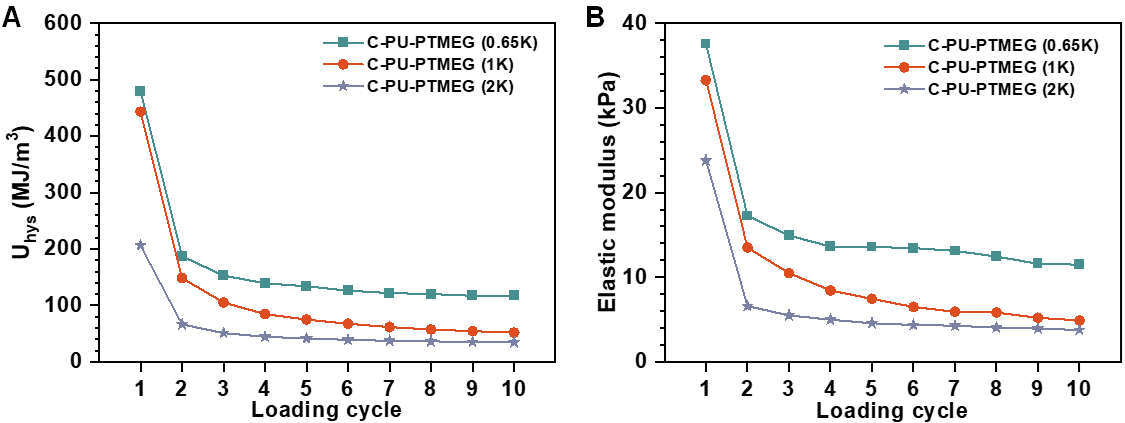
**

**Fig. S6.** (A) Energy loss and (B) change of elastic modulus of C-PU-PTMEGs in successive loading-unloading cycles at a tensile rate of 100 mm min^-1^.

**2.9. Cross-hatch tests**

**
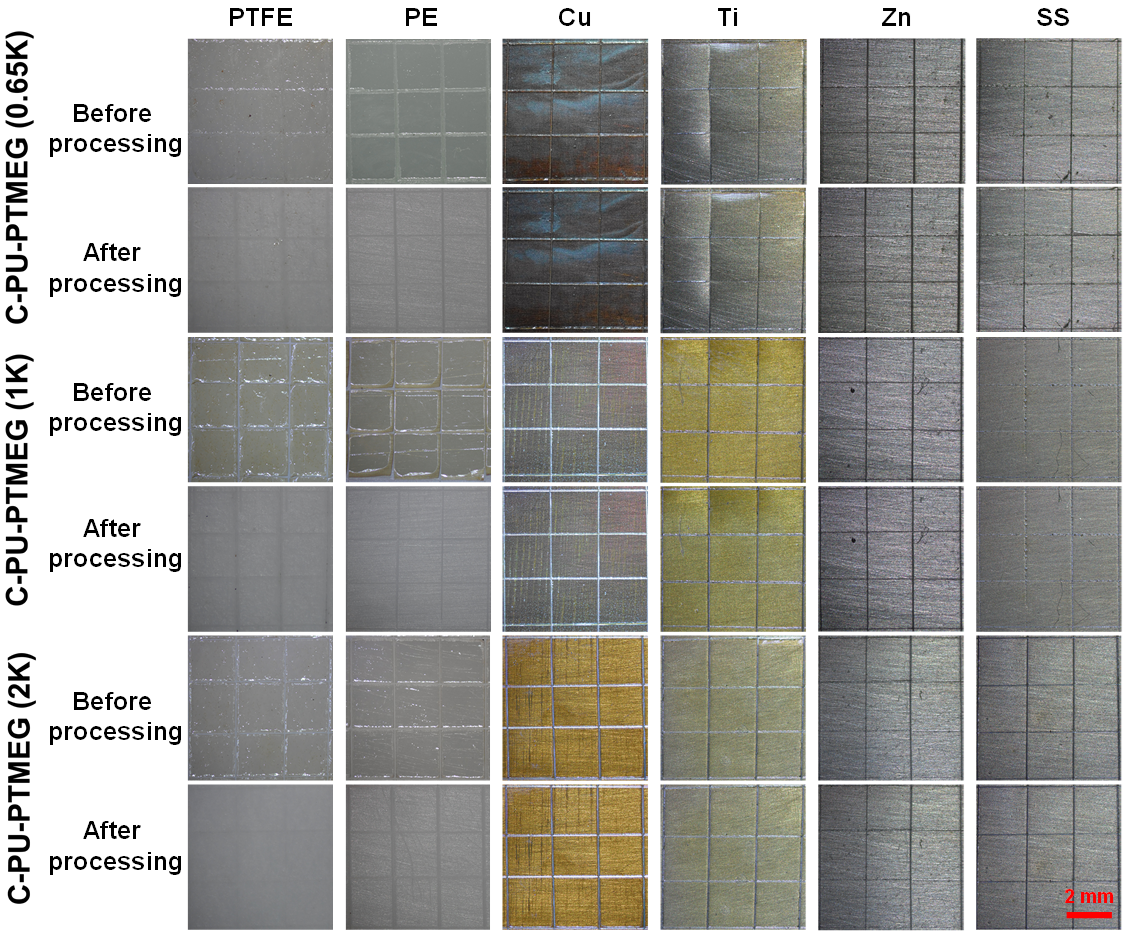
**

**Fig. S7.** Photos of the cross-hatch tests after 5 min of 3 M tape treatment.

**2.10. Weight load test**


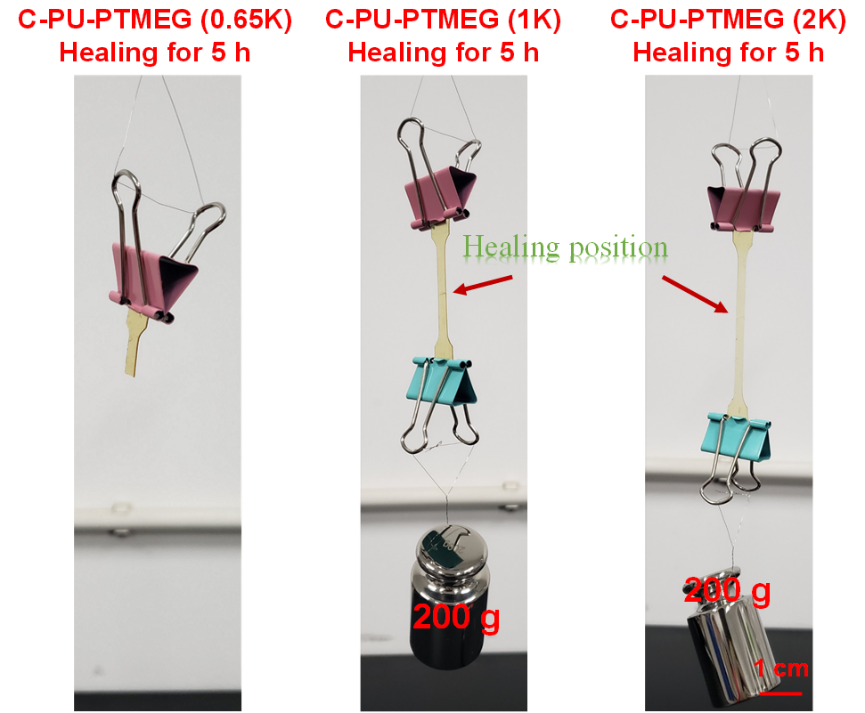


**Fig. S8.** A 200 g weight bearing test was performed when repairing for 5 h at 37 ℃.

**2.11. Swelling property**


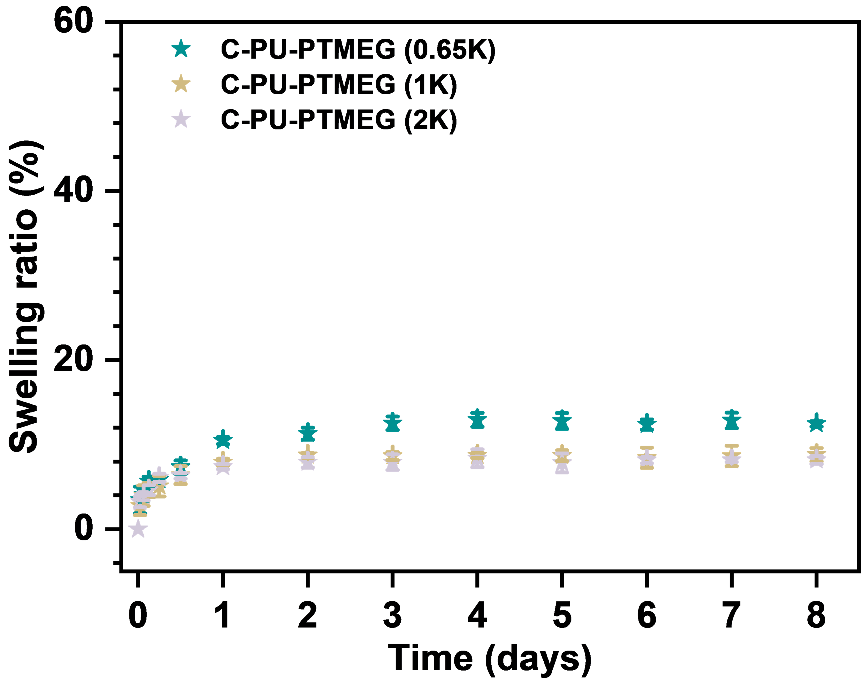


**Fig. S9.** The swelling rate of C-PU-PTMEGs in PBS solution at 37 ℃ for different time.

**2.12. Comparison of mechanical properties of C-PU-PTMEGs**

**
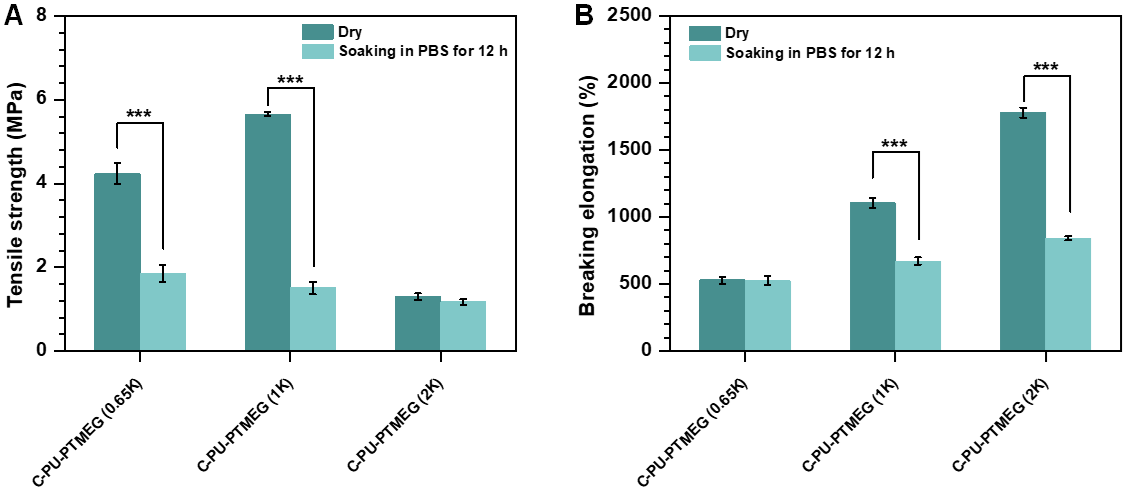
**

**Fig. S10.** Comparison of (A) tensile strength and (B) breaking elongation of C-PU-PTMEGs in dry and wet state.

**2.13. SEM morphology of stents**


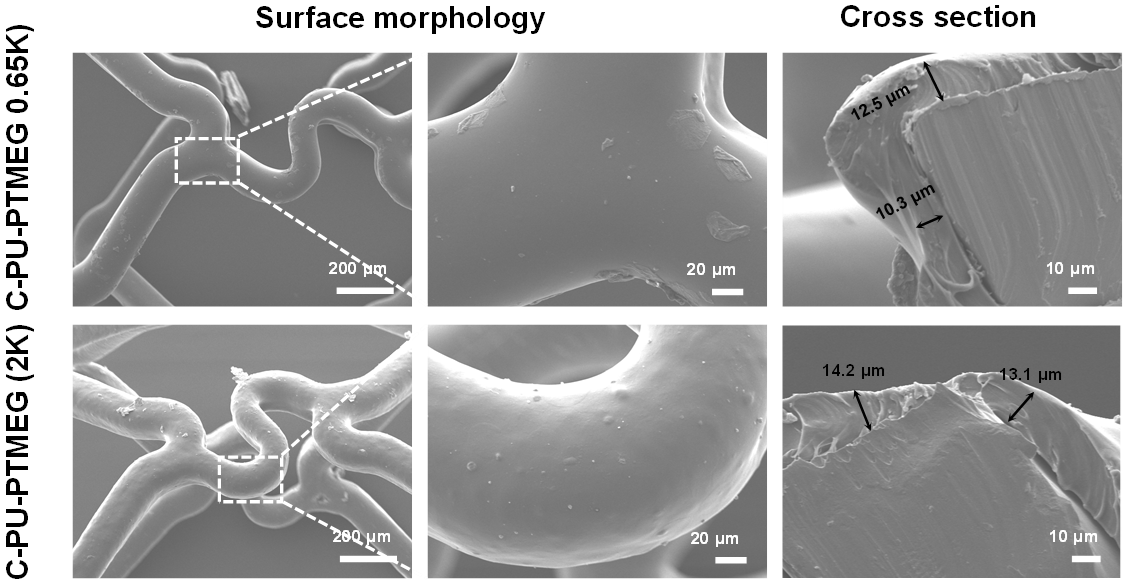


**Fig. S11.** SEM morphology of the vascular stent with C-PU-PTMEG (0.65K) and C-PU-PTMEG (2K) coating after balloon expansion and SEM morphology of the local enlargement in the stent.

**2.14. H&E-stain**

**
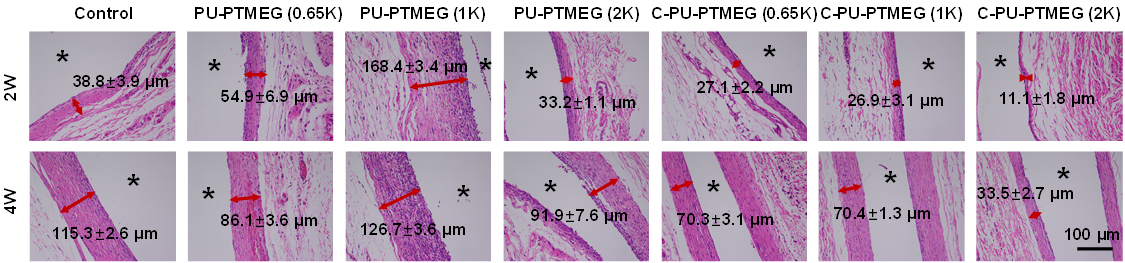
**

**Fig. S12.** Histological and fibrous capsule thickness analysis of inflammatory response within the PU films by H&E-stain. * denotes the implantation location of the polymer films. The red double arrow represents the thickness of the new fibrous capsule.

**2.15. Activation of platelet**


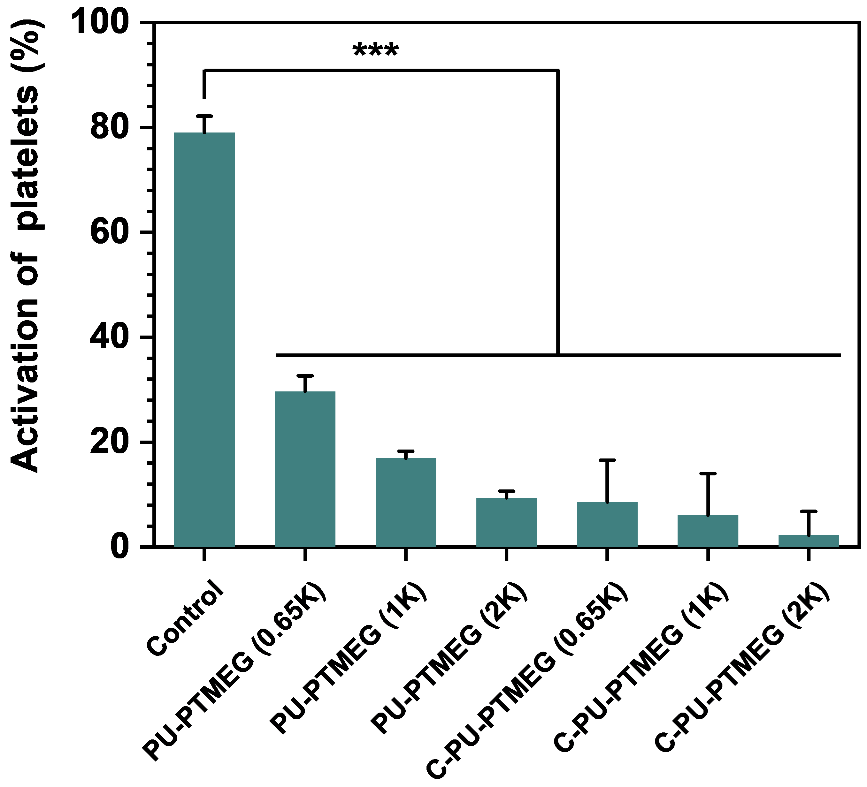


**Fig. S13.** Activation of blood platelets on various samples. Statistically significant was regarded as follows: P<0.001, ***.

**2.16. SEM images of samples**

**
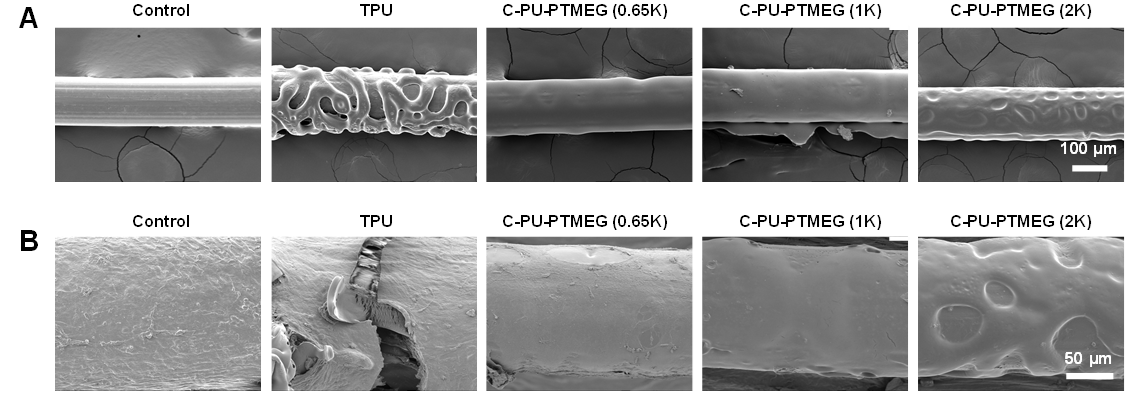
**

**Fig. S14.** SEM images of samples (A) before implantation and (B) after implantation in rat abdominal aorta for 15days.

**References**

[1] P. Kasprzyk, H. Benes, R. K. Donato, and J. Datta, "The role of hydrogen bonding on tuning hard-soft segments in bio-based thermoplastic poly (ether-urethane) s," *Journal of Cleaner Production,* vol. 274, p. 122678, 2020.

[2] A. Niemczyk, A. Piegat, Á. S. Olalla, and M. El Fray, "New approach to evaluate microphase separation in segmented polyurethanes containing carbonate macrodiol," *European Polymer Journal,* vol. 93, pp. 182-191, 2017.

[3] S. Asadpour *et al.*, "Polyurethane-polycaprolactone blend patches: scaffold characterization and cardiomyoblast adhesion, proliferation, and function," *ACS Biomaterials Science & Engineering,* vol. 4, no. 12, pp. 4299-4310, 2018.

[4] Y. Chen, Q. Duan, J. Zhu, H. Liu, L. Chen, and L. Yu, "Anchor and bridge functions of APTES layer on interface between hydrophilic starch films and hydrophobic soyabean oil coating," *Carbohydrate Polymers,* vol. 272, p. 118450, 2021.

[5] C. Wang *et al.*, "Dual-functional anti-biofouling coatings with intrinsic self-healing ability," *Chemical Engineering Journal,* vol. 389, p. 123469, 2020.

[6] X. Zhou *et al.*, "Resveratrol accelerates wound healing by attenuating oxidative stress-induced impairment of cell proliferation and migration," *Burns,* vol. 47, no. 1, pp. 133-139, 2021.
